# Assessment of dynamic stability and identification of key tasks and parameters in patients with unilateral and bilateral vestibulopathy: a laboratory-based study

**DOI:** 10.3389/fnins.2025.1624948

**Published:** 2025-09-08

**Authors:** Gautier Grouvel, Anissa Boutabla, Julie Corre, Romain Bechet, Samuel Cavuscens, Maurizio Ranieri, Jean-François Cugnot, Christopher McCrum, Raymond van de Berg, Nils Guinand, Stéphane Armand, Angélica Pérez Fornos

**Affiliations:** ^1^Division of Otorhinolaryngology Head and Neck Surgery, Geneva University Hospitals and University of Geneva, Geneva, Switzerland; ^2^Kinesiology Laboratory, Geneva University Hospitals and University of Geneva, Geneva, Switzerland; ^3^Research Center of Skeletal Muscle and Movement, Geneva University Hospitals and University of Geneva, Geneva, Switzerland; ^4^Arts et Métiers Institute of Technology, Université Sorbonne Paris Nord, IBHGC – Institut de Biomécanique Humaine Georges Charpak, Paris, France; ^5^Clinical Neurosciences Department, Neurorehabilitation Department, Geneva University Hospitals, Geneva, Switzerland; ^6^Department of Nutrition and Movement Sciences, NUTRIM School of Nutrition and Translational Research in Metabolism, Maastricht University Medical Center+, Maastricht, Netherlands; ^7^Division of Balance Disorders, Department of Otorhinolaryngology and Head and Neck Surgery, Maastricht University Medical Center+, Maastricht, Netherlands

**Keywords:** bilateral vestibulopathy, dynamic stability, imbalance, functional follow-up of patients, short-form FGA, COSMIN domains

## Abstract

Chronic imbalance is the cardinal symptom in bilateral vestibulopathy patients (BV), and in a subset of symptomatic unilateral vestibulopathy patients (UV), leading to a significant impact on their daily lives. Despite these profound effects, such as the risk of falls, the mechanism of imbalance remains complex, posing challenges both for monitoring patients’ functional status and for evaluating rehabilitation therapies. The aim of this study was to assess the dynamic stability of patients with BV and UV during multiple motor tasks and to provide a summary of the most relevant tasks and biomechanical parameters. The purpose was to propose a “short-form FGA” (Functional Gait Assessment) test to reduce the length and complexity of tests, to be able to evaluate future therapies longitudinally, and to monitor functional follow-up of patients. Dynamic stability, spatio-temporal and kinematic parameters were calculated for 10 BV patients, 10 UV patients and 10 asymptomatic controls while walking at three self-selected walking speeds, while performing dual tasks and while completing the 10 tasks of the FGA battery. Two (validity and interpretability) of the four COSMIN domains and clinical applicability were evaluated to identify relevant tasks and parameters to the study population, i.e., good discriminant and convergent validity, and good clinical applicability. The comfortable and slow gait, as well as the turn pivot, eyes closed, and tandem walk tasks were identified as the most relevant for characterizing dynamic stability in these patients. Easily interpretable and visually assessable parameters, such as walking speed, center of mass displacement, step width, trunk movement, stiffness of the head/trunk, and number of steps, were identified as the most relevant. In contrast, stability parameters such as margin of stability or whole body angular momentum did not prove to be effective parameters. These relevant parameters should enable future studies to evaluate rehabilitation therapies such as vestibular implants or physiotherapy, as well as to monitor patients’ functional status. Future studies should validate these results and assess the missing psychometric properties of these parameters.

## 1 Introduction

The vestibular system, located in the inner ear, is a sensory organ that provides unique head-motion information to ensure visual and postural stability during movements (Cullen and Sadeghi, 2008). This information, coupled with vision and proprioception, enables humans to stabilize themselves, even during complex motor tasks (Horlings et al., 2009). Lesions of the vestibular system can occur, and although the etiology may be ototoxic, traumatic, infectious, congenital or genetic, it remains unknown in some cases (Guinand et al., 2012; Van Stiphout et al., 2022). One or both ears can be affected, resulting in unilateral or bilateral vestibulopathy. The most frequent symptom for BV patients and a subset of UV patients is chronic imbalance, which has an impact on their quality of life (Guinand et al., 2012; Lucieer et al., 2020). However, some patients have developed adaptive or compensatory strategies allowing them to perform simple tasks such as walking on flat ground with relative ease (Grouvel et al., 2024) and without visible impairment. Despite this, BV patients and a majority of UV patients have difficulties moving around in dimly lit environment, walking on uneven ground, or making rapid head movements while walking (Lucieer et al., 2020; van de Berg et al., 2015; Cohen et al., 2018).

Other symptoms, such as oscillopsia, have been explored, and previous studies attempted to assess oscillopsia using a quantitative test of dynamic visual acuity (Hillman et al., 1999; Lambert et al., 2010; Guinand et al., 2012; Starkov et al., 2020; Guinand et al., 2016). As far as imbalance is concerned, there are significant gaps in knowledge within this population, particularly concerning stabilization mechanisms, patients’ limiting tasks and the parameters that highlight their difficulties. Despite a growing number of studies focusing on movement analysis in patients with vestibulopathy, dynamic stability was not extensively studied. Dynamic stability refers to the body’s ability to maintain balance while moving, adapting to disturbances and avoiding falls (Hurmuzlu and Basdogan, 1994), while spatio-temporal parameters and kinematics describe how the body moves (Baker, 2013). Studies reported that spatio-temporal parameters are deteriorated in BV (McCrum et al., 2019; Herssens et al., 2020; Boutabla et al., 2025) and UV (Boutabla et al., 2025; Majerník and Molčan, 2018) patients compared to an asymptomatic population. In a previous study (Grouvel et al., 2024), conventional kinematic analyses, such as joint and segment kinematics, were found to provide limited insight into patients’ movement patterns. To better distinguish between pathological and asymptomatic groups, it is essential to consider more specific parameters, such as movement variability assessed with GaitSD (Grouvel et al., 2024; Sangeux et al., 2016), which summarizes a subject’s kinematic variability during walking in a single number, with higher values indicating less stable and more irregular gait. Another relevant parameter is the head anchoring index (Grouvel et al., 2024; Assaiante and Amblard, 1993; Schreiber et al., 2016), which measures head movements, and reflects the head stabilization strategies adopted by the participant. Moreover, these parameters can provide valuable indications of disease severity, as they can be used to monitor the patient’s functional status over time.

A better understanding of the mechanisms of dynamic stabilization in patients with vestibulopathy is essential to improve diagnosis, to develop specific, precise, rapid and quantitative assessments of balance function, and to optimize rehabilitation strategies. This would enable to validate future therapies and functional follow up of patients, therefore filling a major gap in vestibular medicine, i.e., the longitudinal monitoring of disease progression. To our knowledge, existing studies only examined walking under different walking speed conditions (slow, comfortable, or fast) (Boutabla et al., 2025; Liu et al., 2017; Herssens et al., 2021). To better assess the challenges faced by these patients, their movement should be analyzed during dynamic tasks that impose greater postural demands, particularly under conditions that limit sensory inputs from other modalities. After all, the complaint of imbalance in this population manifests itself mainly in complex environments, such as low-light spaces or uneven surfaces. As a result, these measures would offer a more accurate representation of the difficulties encountered by patients.

To fill these gaps, we analyzed a set of parameters during dynamic tasks in populations with BV and UV and compared them with an asymptomatic group. The tasks selected included walking and double-task walking, as well as items from the Functional Gait Assessment (FGA) (Wrisley and Kumar, 2010) which are representative of patients’ daily lives and may correspond to situations that limit the compensation of other sensory inputs, e.g., gait with eyes closed.

The aim of this study was to propose a simplified version of the FGA for rapid and accurate assessment of patients. To achieve this, the dynamic stability in patients with BV and UV was investigated. We looked into which tasks and biomechanical parameters were most discriminative to distinguish BV and UV patients from asymptomatic controls (HS). Furthermore, the correlation of these tasks and parameters with quantified symptomatology was explored, in addition to their clinical applicability (Villafañe et al., 2016). Clinical applicability is defined as the ability to assess task-related parameters in everyday clinical practice, without the need for complex tools and specialists. Given that patients with vestibulopathy often develop compensatory strategies over time, we hypothesized that significant differences would be observed in tasks where the opportunity for compensation using other sensory inputs was limited, such as walking with eyes closed. For the parameter analysis, we anticipated that the patient groups would demonstrate greater mechanical stability in coping with their imbalance, as indicated by greater margins of stability and larger amplitudes of center of mass, compared to asymptomatic controls. However, considering that patient instability can often be transient, we did not expect to observe consistent or large differences across all tasks and parameters assessed. The findings of this study could facilitate the development of more targeted tests and analyses for future clinical evaluations and interventions.

## 2 Materials and methods

### 2.1 Population

Ten patients (5 females, median [Interquartile range: IQR], age: 64.4 [9.6] years, height: 1.64 [0.09] m, weight: 71.4 [10.6] kg, BMI: 26.4 [3.8] kg/m^2^) with chronic bilateral vestibulopathy (BV), 10 patients (5 females, 4 affected on the left side, median [IQR], age: 63.4 [6.2] years, height: 1.67 [0.16] m, weight: 79.6 [17.4] kg, BMI: 27.9 [2.4] kg/m^2^) with chronic unilateral vestibulopathy (UV), and 10 asymptomatic controls (HS) (6 females, median [IQR], age: 64.6 [10.0] years, height: 1.71 [0.07] m, weight: 70.9 [12.7] kg, BMI: 24.2 [3.6] kg/m^2^) took part in this study (Table 1). BV patients were recruited according to the guidelines of the Classification Committee of the Bárány Society (Strupp et al., 2017): unsteadiness when walking or standing, oscillopsia and/or worsening of imbalance in darkness/uneven ground, no symptoms while sitting or lying down, bilaterally reduced or absent vestibulo-ocular reflex documented by a caloric test, video-head impulse test (vHIT), or torsion swing test, and finally not better accounted for by another disease. These strict inclusion criteria ensured that the patient population recruited corresponded to patients with severe BV. Regarding the UV patients, they had to have a deficit for at least 3 months and to meet clinical vHIT requirements, with gain values below 0.6 for the lateral semicircular canals of the affected ear, and to have a normal vestibular function in the other ear (vHIT gain values above 0.8). Finally, all HS met a criterion of normal vHIT gain values for all semicircular canals (vHIT gain values above 0.8). All study participants were over 18 years of age and provided their written informed consent. The study was designed and conducted in accordance with the guidelines of the Declaration of Helsinki and was approved by the Cantonal Commission for Research Ethics of Geneva (NAC 11-080 CER 11-219).

**Table 1:** Etiological data for participants of the study, patient reported outcomes (DHI score), and FGA detailed and total scores: the lower the score, the more pathological the patient (Wrisley and Kumar, 2010).

Participants	Sex	Affected side	Etiology	DHI		FGA											
				Score	Median (Q1–Q3)	Gait level surface	Change speed	Horizontal head turns	Vertical head turns	Turn pivot	Step obstacle	Tandem walk	Eyes closed	Backwards	Steps	Total	Median (Q1–Q3)
BV																	
1	F	Both	Ototoxic	48	40 (20–48)	3	3	3	3	2	1	0	0	2	2	19	20.5 (19.3–20.5)
2	F	Both	Genetic	46		2	2	3	3	2	3	0	1	2	3	21	
3	M	Both	Idiopathic	12		2	3	3	1	3	3	0	1	1	3	20	
4	F	Both	Idiopathic	34		2	3	2	2	3	3	0	1	3	2	21	
5	F	Both	Idiopathic	20		3	2	2	2	3	3	0	0	2	3	20	
6	F	Both	Idiopathic	74		3	3	2	2	2	2	0	1	0	3	18	
7	M	Both	Schwannoma	2		3	3	3	3	3	3	0	0	3	3	24	
8	M	Both	Idiopathic	40		3	3	3	3	3	3	0	2	3	3	26	
9	M	Both	Idiopathic	48		3	3	2	2	2	1	0	0	1	1	15	
10	F	Both	Idiopathic	UN		2	3	2	3	3	2	0	2	3	2	22	
UV																	
1	F	Left	Idiopathic	68	20 (9.5–58)	1	2	1	2	0	2	2	0	2	2	14	27.5 (26.3–27.5)
2	M	Left	Idiopathic	6		3	3	3	3	3	3	3	3	3	3	30	
3	M	Left	Post-labyrinthectomy	64		2	3	3	3	2	3	2	1	2	1	22	
4	F	Right	Idiopathic	14		2	3	3	3	3	3	3	2	3	3	28	
5	M	Left	Schwannoma	20		3	3	3	3	3	3	3	2	3	3	29	
6	M	Right	Idiopathic	8		3	3	3	3	3	3	2	1	3	3	27	
7	F	Right	Idiopathic	2		3	3	3	3	3	3	3	1	3	3	28	
8	M	Right	Traumatic	11		3	3	3	3	2	3	3	2	3	3	28	
9	F	Right	Schwannoma	52		3	2	3	3	2	2	2	3	3	3	26	
10	F	Right	Idiopathic	22		3	3	2	2	3	3	3	2	3	3	27	
HS																	
1	F	NA	NA	NA	–	3	3	3	3	3	3	3	2	3	3	29	29 (29–29)
2	F	NA	NA	NA		3	3	3	3	3	3	3	3	3	3	30	
3	M	NA	NA	NA		3	3	2	3	3	3	3	2	3	3	28	
4	M	NA	NA	NA		3	3	3	3	3	3	3	3	3	3	30	
5	F	NA	NA	NA		3	3	3	3	3	3	3	3	3	3	30	
6	F	NA	NA	NA		3	3	3	3	3	3	3	3	3	3	30	
7	M	NA	NA	NA		3	3	3	3	3	3	2	3	3	3	29	
8	F	NA	NA	NA		3	3	3	3	3	3	3	2	3	3	29	
9	M	NA	NA	NA		3	3	3	3	3	3	3	2	3	3	29	
10	M	NA	NA	NA		3	3	3	3	3	3	2	2	2	3	27	

BV, Bilateral vestibulopathy patients; UV, Unilateral vestibulopathy patients; DHI, Dizziness Handicap Inventory; FGA, Functional Gait Assessment; F, Female; M, Male; NA, Not Applicable; UN, Unavailable.

### 2.2 Equipment and protocol

The 3-dimensional movements of the participants were measured using a 12-camera optoelectronic system (Oqus7+, Qualisys, Göteborg, Sweden), set at a 100 Hz sampling frequency. The measurements were acquired at the Kinesiology laboratory of Geneva University Hospitals. Reflective markers (14 mm diameter) were placed on body anatomical landmarks according to the Conventional Gait Model (CGM) 1.0 (Davis et al., 1991; Leboeuf et al., 2019).

The protocol measurement started with a 10-s recording of the participants standing upright, useful for model calibration during data processing. Then, participants were asked to perform three walking trials at each self-selected walking speeds: comfortable, slow, and fast. The measurements continued with two dual-task recordings during which participants had to walk at a comfortable walking speed and quote animal names and words beginning with the letter p. Finally, the participants performed a set of tasks extracted from the Functional Gait Assessment (FGA) (Wrisley and Kumar, 2010). Details of the tasks and the number of repetitions performed are shown in Table 2.

**Table 2:** Description of tasks performed during the measurement protocol.

Tasks	Description	Number of recordings
Comfortable gait	Walking at a comfortable speed	3
Slow gait	Walking at a slow speed (compared to the comfortable gait)	3
Fast gait	Walking at the fastest possible speed (without running)	3
Double task—Animal	Walking and quoting the most animal names	1
Double task—Letter	Walking and quoting the most words beginning with the letter p	1
Change speed*	Walk comfortably, quickly, then slowly	1
Horizontal head turns*	Walking and alternating horizontal rotations (from left to right) of the head	1
Vertical head turns*	Walking and alternating vertical rotations (up and down) of the head	1
Turn pivot*	Walk comfortably, then turn around and stop	1
Step obstacle*	Walk comfortably over an obstacle	1
Tandem walk*	Walking on a narrow base of support (heel toe on a line)	1
Eyes closed*	Walking with eyes closed	1
Backwards*	Walking backwards	1
Steps*	Going up and down stairs	1

*Tasks included in the FGA.

### 2.3 Data processing

The 3-dimensional marker trajectories were labeled using Qualisys Track Manager software (QTM 2019.3, Qualisys, Göteborg, Sweden) and exported in the C3D file format.^1^ All data processing was performed using Matlab (R2022b, The MathWorks, Natick, MA, United States) with the C3D parser from the Biomechanics Toolkit (BTK) (Barre and Armand, 2014). The marker trajectories were interpolated to fill gaps in the data using a reconstruction method that relies on marker inter-correlations (Gløersen and Federolf, 2016). Then, marker trajectories were filtered using a 4th order low-pass Butterworth filter with a cut-off frequency set at 6 Hz. In each trial file, virtual calculated markers were added to define joint centers of the upper and lower limbs (Hara et al., 2016; Joint Center Calculations, n.d.) and the center of the posterior and anterior iliac spines. Upper and lower limb segment coordinate systems were also included in the C3D file as virtual markers, to facilitate parameter calculations. Gait events, i.e., left and right foot strikes and foot offs, were automatically detected using a custom-made algorithm in Matlab (Fonseca et al., 2022). To prevent potential detection errors, each event was visually checked and corrected if needed by an operator. All the processed data are available in an online repository, see Data Availability section.

### 2.4 Data analysis

Nineteen parameters describing patient movement and stability were calculated and compared. These outcomes were grouped into four categories: dynamic stability, spatial, temporal, and kinematic. Parameters were calculated for all the tasks except for the tandem walk, backwards, and steps tasks where only one parameter was calculated due to poor recording quality and the difficulty of labeling markers. When parameters could be calculated for both left- and right-side gait cycles, such as margin of stability, step width, or whole-body angular momentum, we arbitrarily took the left side for BV patients and HS participants (as no significant differences between the two sides were found), and the pathological side for UV patients. When applicable, the parameters were calculated for the foot strike of the leading foot. When several parameter values were calculated for each gait cycles of the trial, the median value was extracted.

**Dynamic stability parameters**

**Figure 1:**
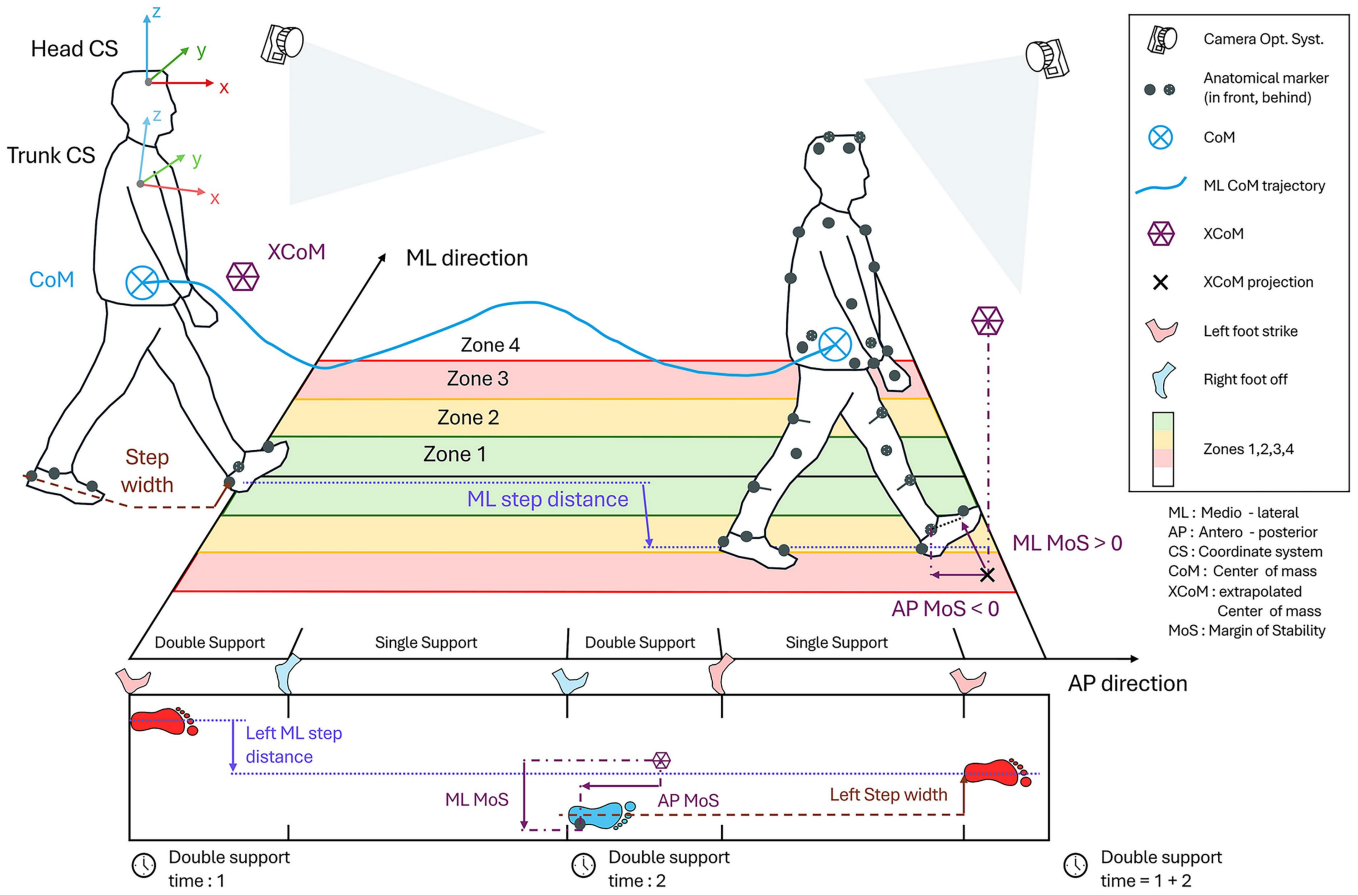
Measurement settings and description of calculated parameters.

- Center of mass (CoM)—Medio-lateral range of motion (ML CoM rom) (Figure 1): CoM movement during locomotion helps to better understand the underlying mechanisms of gait imbalance and assess the risk of falls in patients (Lugade et al., 2011). The CoM was calculated using 13 markers to reconstruct 9 body segments as developed by Tisserand et al. (2016). This method, which provided accurate estimates compared to a full-body method, was feasible with the marker set used.
- Margin of Stability (MoS)—Medio-lateral and antero-posterior (Figure 1): The MoS (Herssens et al., 2021; Hof et al., 2005; Curtze et al., 2024) was calculated in the sagittal and frontal planes using the following formula:
MoS=BoS−xCoM

Where the base of support (BoS) was defined as the lateral malleolar marker of the leading foot (Hak et al., 2013), and the extrapolated center of mass (xCoM) was defined using the vertical projection of the CoM and the velocity of the whole-body CoM (Hof, 2008).

- Whole body angular momentum (WBAM) – Range of sagittal, coronal, and frontal planes: WBAM represents the rotational behavior of the whole-body and is therefore a relevant measure for assessing dynamic balance during walking (Herr and Popovic, 2008; Silverman et al., 2012; Bennett et al., 2010). This parameter was calculated as the sum of the segment angular momenta of the 13 segments whole-body model, each transferred to the whole-body CoM (Herr and Popovic, 2008; Negishi and Ogihara, 2023).

**Spatial parameters**

- CoM score (Figure 1): This parameter is similar to the CoM range of motion, but instead of taking patient movement into account, four medio-lateral zones have been virtually defined on the floor – inspired by FGA scoring (Wrisley and Kumar, 2010), and we measured the number of times the center of mass passes through each zone. The more the CoM passes through the outer zones, the higher the score. This score attempts to reproduce the method used by the Geneva Balance Test (GBT) developed by our group (Monin et al., 2023), and would make it possible to identify patients with deviations and catch-ups in their gait, i.e., normal gait but sudden instability with a catch-up step and large movements that cause the CoM to move away from its original position. This score is calculated for the entire trial.
- Foot score (Figure 1): This parameter is similar to the previous one, but instead of measuring the time the CoM is in each zone, we counted the time both feet are in the zones. With this parameter, we are once again seeking to highlight the deviations and catch-ups, throughout the trial.
- Step width (Figure 1): Step width is calculated as the distance between the heels of the left and right foot at the foot strike of the leading foot.
- Medio-lateral step distance (ML step) (Figure 1): This parameter corresponds to the maximum medio-lateral distance between two ipsilateral steps. We have not found any other studies that have looked at a similar measure. This parameter is derived from video observations in which bilateral vestibulopathy patients are seen regularly taking steps to the side to restabilize themselves, without their gait being deviated.
- Step number: This is the number of steps participants are able to perform during the tandem walk task, according to the FGA scoring system (Wrisley and Kumar, 2010).

**Temporal parameters**

- Walking speed: For each walking task the walking speed was calculated.
- Double support time (Figure 1): This parameter corresponds to the time when both feet are on the ground during a gait cycle.
- Task time: It corresponds to the time needed for the participant to perform the task.

**Kinematic parameters**

- Head Anchoring Index (Head AI)—pitch, roll, yaw planes (Figure 1): A positive Head AI indicates a stabilization strategy in space (i.e., head and trunk movements are independent), and a negative value indicates a stabilization strategy on the trunk (i.e., head and trunk move in “block”). The Head AI was calculated based on the standard deviation of the head orientation in the global coordinate system and on the standard deviation of the head orientation relative to the trunk movement (Grouvel et al., 2024; Assaiante and Amblard, 1993; Schreiber et al., 2016).
- Head angular velocity: The root mean square (RMS) of the normalized head angular velocity was calculated to analyze the amount of movement of the head during the tasks.
- Trunk angular velocity: The RMS (of the normalized trunk angular velocity was calculated to analyze the amount of movement of the trunk during the tasks).
- Gait Standard Deviation (GaitSD): This parameter summarizes the participant variability during movement into a single value. The higher the value, the greater the participant’s variability during movement. The GaitSD was calculated as the square root of the average variance of 9 kinematic variables (Sangeux et al., 2016).

### 2.5 Statistical analysis

**Figure 2:**
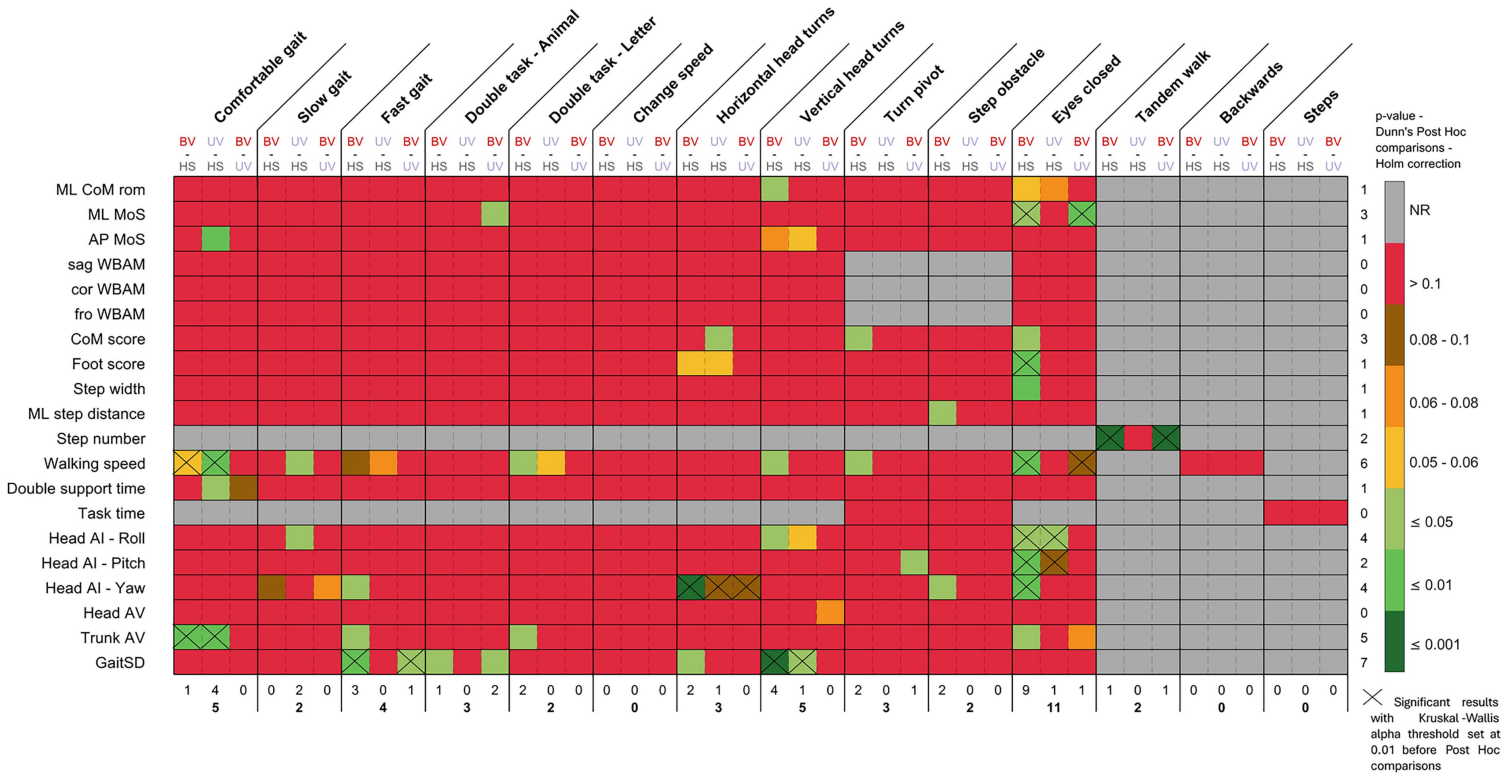
Dunn’s *post-hoc* statistical test with Holm correction for all the calculated parameters for each task (after Kruskal-Wallis test with alpha set at 0.05). X: Significant results with Kruskal-Wallis alpha threshold set at 0.01 before *Post Hoc* comparisons. NR: Not relevant—statistical test not performed as no data was calculated. Color scale—from red to yellow: *p*-values were not significant but allow to see the distribution of statistical results; light green: *p*-value ≤ 0.05; green: *p*-value ≤ 0.01; dark green: *p*-value ≤ 0.001. A line corresponds to a parameter. A column represents a task. A sub-column represents the comparison between groups (BV-HS; UV-HS; BV-UV).

**Figure 3:**
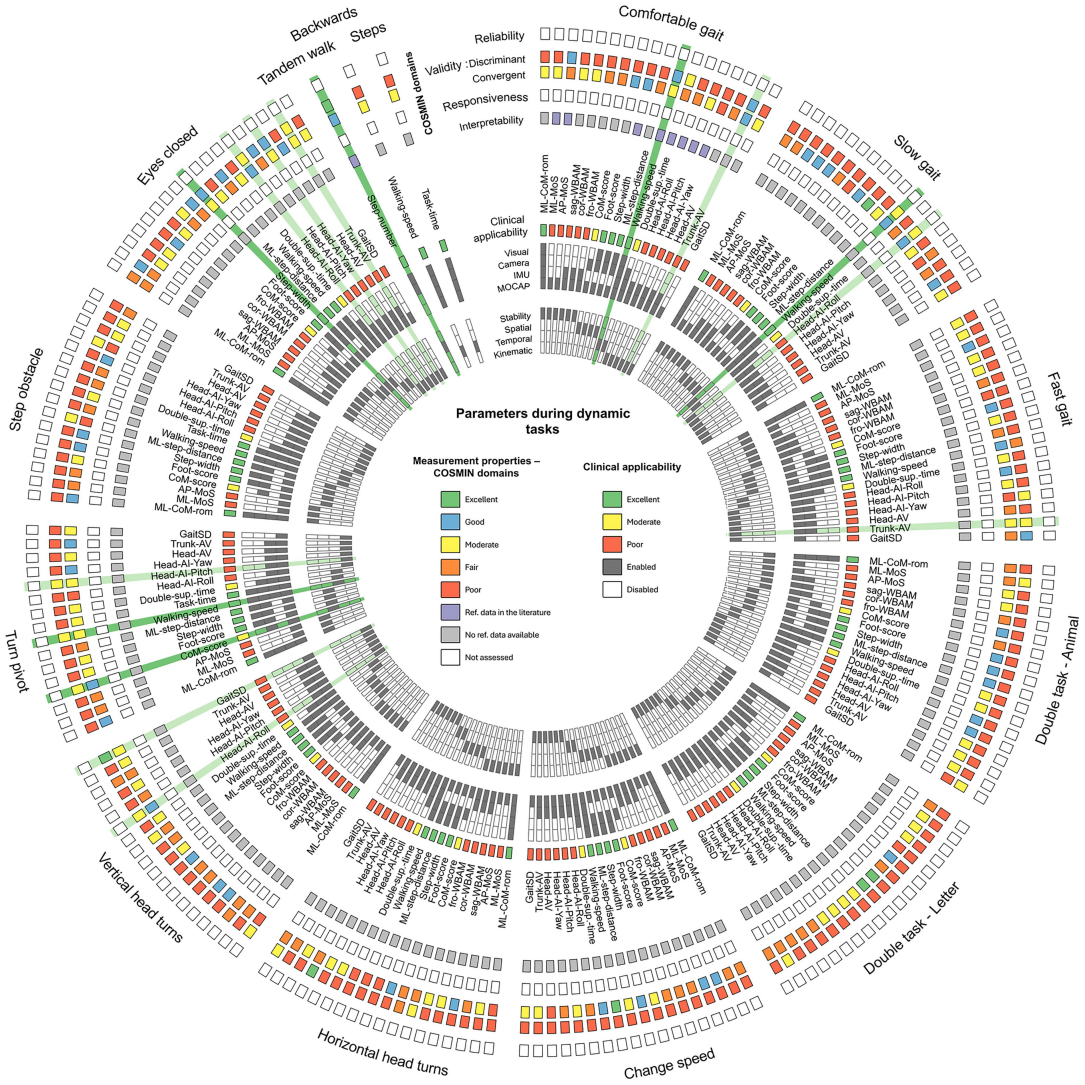
Circos plot (Krzywinski et al., 2009) synthesizing the main characteristics and measurement properties of each parameter for each task. Relevant parameters are highlighted in dark green when at least the discriminant and convergent validity are moderate, and the clinical applicability is excellent. Other interesting parameters are highlighted in light green when at least the validity is moderate, and the clinical applicability is poor. Measurement properties—Green: Excellent discriminant validity (*p*-values ≤ 0.001) or Excellent correlation (*r* > 0.8)—Blue: Good discriminant validity (*p*-values ≤ 0.01) or Good correlation (0.6 < *r* ≤ 0.8)—Yellow: Moderate discriminant validity (*p*-values ≤ 0.05) or Moderate correlation (0.4 < *r* ≤ 0.6)—Orange: Fair discriminant validity (0.05 < *p*-values < 0.1) or Fair correlation (0.2 < *r* ≤ 0.4)—Red: Poor discriminant validity (*p*-values ≥ 0.1) or Poor correlation (*r* ≤ 0.2)—Purple: reference data can be found in the literature—Gray: no reference data can be found in the literature—White: parameter not assessed. Clinical applicability—Green: Excellent clinical applicability (parameter easy to assess with the eyes or camera, and easy to interpret)—Yellow: Moderate clinical applicability (parameter easy to assess, but requires some knowledge of human motion analysis)—Red: Poor clinical applicability (parameter difficult to assess, and difficult to interpret in a clinical environment)—Dark gray: associated category checked—White: associated category unchecked.

Given the small number of participants in each group (10 participants) and the non-normality of the majority of the data (evaluated with a Shapiro–Wilk test), we performed non-parametric statistical tests. To evaluate and compare the parameters calculated for each task, two of the four COSMIN domains (Mokkink et al., 2010) were analyzed: validity (Figure 2) and interpretability (Figure 3). The reliability, and responsiveness were not assessed in this study, (no data were collected), but are represented in the Figure 3. The clinical applicability of the parameters was also estimated by looking at the ease with which the parameter could be evaluated in everyday clinical practice, its representation of dynamic stability, and its classification according to calculation complexity, from the most complex to the simplest. This assessment inherently involved a degree of subjectivity on the part of the authors. To summarize and highlight the relevant tasks and parameters, i.e., good discriminant and convergent validity, and good clinical applicability, a Circos plot (Krzywinski et al., 2009) was generated (Figure 3), which allows visual representation of all results at once. In the Circos plot, the parameters evaluated are arranged in columns around the circumference of the circle. Each parameter is repeated for each task, itself represented around the circle. The rows correspond to the assessment domains or categories associated with the parameters. The colors used in the cells are explained in the legend at the center of the plot. To interpret the results of a parameter for a specific task, follow the corresponding column from the outer edge to the center of the circle.

For discriminant validity, we performed a Kruskal-Wallis test between the three groups. Each parameter was compared between the three groups and for each task. When significant differences appeared, i.e., *p*-value of the Kruskal-Wallis test ≤ 0.05, a *post-hoc* Dunn test (with Holm correction) was used to identify specific between-group differences. However, as the aim of this study was to give recommendations, and due to the large number of Kruskal-Wallis tests carried out, we decided to add the information if the Kruskal-Wallis alpha threshold was 0.01 to reduce the risk of false positives and to provide stronger evidence. Given the restricted sample size, it was determined that stricter adjustments such as the Bonferroni would be too conservative.

For convergent validity, correlations between objective parameters and the DHI score (Jacobson and Newman, 1990) (subjective and clinical score) were calculated. Pearson correlations were used.

For interpretability, we have extracted information from the literature.

For clinical applicability, two key categories were assessed. The first one concerns parameter evaluation tools. All the calculated parameters of this study can be assessed, from the most complex to the simplest, using a motion capture system (MOCAP), inertial sensors (IMUs), a camera, or visual observation. Parameters assessed with simpler tools are easier to apply in the clinic. Afterwards, we estimated the complexity of parameter interpretation and categorized them by their type (stability, spatial, temporal, kinematic). If a parameter was easy to measure, highly representative of dynamic stability, and easy to interpret (e.g., step width or center of mass movement), clinical applicability was considered good. If the parameter was difficult to measure, harder to interpret but still related to stability (e.g., WBAM, GaitSD), clinical applicability was considered moderate to poor.

## 3 Results

### 3.1 Discriminant validity

Figure 2 shows the significant differences between groups for each parameter and task. All statistical test results are shown in Supplementary Data 1. The task with the most significant differences for the calculated parameters was the eyes closed task, with 9 significant differences between BV-HS, 1 significant difference between UV-HS and BV-UV. Other tasks that discriminated between groups were the comfortable gait task (5 significant differences: 1 BV-HS, 4 UV-HS), as well as the vertical head turns tasks with 5 significant differences (4 BV-HS, 1 UV-HS).

The most discriminating parameter between groups, all tasks combined, was the GaitSD, with 7 significant differences. For example, in the vertical head turns task, significant differences were observed between BV-HS (*p* < 0.001), and between UV-HS (*p* = 0.017). Following this, the Walking speed parameter exhibited 6 significant differences. Trunk AV, Head AI—Roll and—Yaw have, respectively, 5, 4 and 4 differences, and finally ML MoS and CoM scores had 3 differences.

Additionally, the Tandem Walk task revealed a significantly lower number of steps for BV, BV-HS (*p* < 0.001) and BV-UV (*p* < 0.001).

### 3.2 Convergent validity

The correlations between the objective parameters and the DHI score are highlighted in Figure 3, and a heatmap of Pearson’s r correlations is provided in Supplementary Data 2.

For the BV group, the parameters strongly correlated with the DHI score were: the Walking speed for the Change speed task (*r* = −0.81); the Head AI—Roll for the Eyes closed task (*r* = 0.78); the CoM score, the Trunk AV, and the Head AI—Yaw for the Turn pivot task (*r* = 0.75, *r* = −0.74, *r* = 0.71, respectively).

For the UV group, the parameters with a high correlation were: the Foot score (*r* = 0.93), and the Step width (*r* = 0.85) for the Double task (Letter); the AP MoS (*r* = −0.77), the Step Width (*r* = 0.81) for the Slow gait task; and the Step width (*r* = 0.79) for the Comfortable gait task.

### 3.3 Other COSMIN domains

Reliability and responsiveness were not assessed as no data was acquired during this study (Figure 3). Interpretability (MCID) was assessed in the literature for only the Walking speed parameter for the Comfortable gait task (Wellons et al., 2022).

### 3.4 Clinical applicability

The clinical applicability of parameters (Figure 3) is identical from one task to the next. For example, the clinical applicability of the ML CoM rom is the same for the comfortable walking task and the Eyes closed walking task. The ML CoM rom was found to be the best parameter for representing patient stability, and is also easily assessable by video recording or direct visual observation. Other parameters, including the Foot score, Step width, ML step distance and Walking speed, were also found to be relevant in representing patients’ spatio-temporal parameters, and are also easy to assess. By “easily assessable,” we mean parameters that do not require specialized equipment and that can be estimated by clinicians and non-specialists. Finally, CoM score and Double support time appeared to be interesting parameters, but require slightly more precise evaluation tools such as cameras or IMUs.

### 3.5 Data synthesis

As the majority of parameters were calculated and evaluated in this study for the first time (to the best of our knowledge), we highlighted parameters that are relevant for all the tasks.

Parameters were classified as relevant when they:

1. Discriminated between groups (from moderate to excellent),
2. Correlated with the DHI score (from moderate to excellent), and
3. Showed a good Clinical Applicability

The parameters highlighted (in dark green in the Circos Plot of Figure 3) are the Walking speed for the Comfortable gait task; the Walking speed for the Slow gait task; the CoM score and the Walking speed for the Turn pivot task; the Step width for the Eyes Closed task; and finally the number of steps for the Tandem Walk task.

Other parameters of interest but less relevant due to their poor Clinical Applicability have been highlighted in light green (Figure 3), such as Trunk AV, Head AI, or GaitSD.

## 4 Discussion

This study aimed to provide insights into the dynamic stability of vestibulopathy patients compared to an asymptomatic group of participants. Several dynamic stability, spatial, temporal, and kinematic parameters were analyzed for a set of dynamic tasks, mostly extracted from the Functional Gait Assessment (Wrisley and Kumar, 2010). Overall, the results suggested that patients with BV exhibited several parameters significantly different from asymptomatic controls, and that these parameters also correlated with the DHI score (Jacobson and Newman, 1990). These parameters included Walking speed, Center of Mass displacement, Step width and Number of steps (for the Tandem walk task). The most relevant tasks to highlight these differences and correlations were the Comfortable and Slow self-selected walking speed gaits, the Turn pivot, the Eyes closed and the Tandem walk tasks. Even more certainty can be provided for the following results where the Kruskal-Wallis test was more restrictive: the Walking speed and Trunk movements for the Comfortable gait task, the Head movements for the Eyes closed task, and the Number of steps for the Tandem walk task. Other parameters and tasks could have been highlighted. However, as the tasks did not present parameters with good clinical applicability, we decided not to include them in the main results.

These results are in line with the initial hypothesis that tasks limiting compensatory sensory input may be the most discriminating tasks. The results emphasize the critical role of the vestibular system in maintaining dynamic balance (Horlings et al., 2009), showing that patients who rely on compensatory strategies, such as vision or proprioception, are particularly affected when these sensory inputs are restricted. Although vision deprivation was simulated by closing the eyes, it would be relevant to repeat these measurements in a dark environment, where the light intensity is controlled. With regard to proprioception, no task that actually impair proprioception was evaluated. However, it has been shown that proprioceptive vestibular rehabilitation was beneficial for the treatment of patients with peripheral vestibular hypofunction, compared to a group without proprioceptive rehabilitation (Özaltın et al., 2024). These conditions would therefore appear to be relevant to a better assessment of the impact of the vestibular system on the maintenance of balance. Caution is called for: the extent to which these parameters consistently reflect vestibular deficits in larger populations, this remains to be validated. Moreover, fewer parameters were discriminant for UV patients compared to the HS group. This might suggest that a single vestibular system, i.e., one functional inner ear, can already provide a “relatively useful” functional status. In other words, therapies aimed at restoring the function of at least one ear, e.g., an unilateral vestibular implant (Guinand et al., 2015), could help resolve a considerable part of the symptoms related to imbalance. Nevertheless, this assumption should be interpreted cautiously, as the functional status of UV patients is likely influenced by a complex interplay of factors beyond vestibular input alone.

Walking speed proved to be a potential discriminating parameter between groups in the Slow gait task, with significantly reduced values in patients with vestibulopathy. This reduction in speed may be explained by the fact that the task is more difficult to perform in the absence of a functional vestibular system. Patients tend to rely more on visual and proprioceptive afferents, and slowing gait helps to integrate the different inputs. Furthermore, we note that a small proportion of patients with bilateral deficits tend to increase their walking speed during this task in order to minimize the time spent balancing on one foot during the single stance phase. This might indicate that the contribution of the vestibular system to locomotor postural control is significant, but becomes progressively less decisive as gait speed increases (Boutabla et al., 2025; Brandt et al., 1999; Fitzpatrick et al., 1999; Dakin et al., 2013; Forbes et al., 2017).

FGA tasks are widely used to assess patients’ postural stability during dynamic movements. Although this test is an enhancement of the 8-item Dynamic Gait Index (DGI) (Shumway-Cook and Woollacott, 2001), its overall duration and the usefulness of the whole assessment battery for patients with vestibulopathy seem questionable. The FGA includes the Eyes closed walking task, the tandem walk and the backward tasks, which can increase patient fatigue and potentially affect the reliability of results. Based on the results of this study, the Eyes closed, and Tandem walk tasks were found to be particularly relevant for distinguishing differences between patients and asymptomatic participants. In addition, the detailed FGA task score of this study is the lowest for the Tandem walk and Eyes closed tasks for both patient groups (Table 1) with a majority score of 0 out of 3. It might therefore be interesting to focus only on these two FGA tasks to assess patients’ dynamic stability. Furthermore, these tasks are easily implementable in a standard clinical environment without the need for specialized equipment to record patient movements. Both tasks also represent real-life situations commonly encountered by patients, such as walking in the dark or walking along a narrow sidewalk. Further validation in larger cohorts will be essential to validate task reduction in clinical practice.

Regarding the Turn pivot task which showed promising results, there were not a large number of parameters that discriminate between groups, but the results mostly correlated with patients’ subjective outcomes, the DHI score. It might still be appropriate to include this task in a future functional test. Indeed, when performing this task, patients seem to adopt different strategies while turning and stabilizing. Walking speed during this task will also be significantly impaired.

In this study, we aimed to highlight dynamic stability parameters such as MoS, WBAM and displacement of the CoM, to understand patients’ stabilization mechanisms and highlight limiting tasks. For MoS, significant differences between BV and HS were observable only for the Eyes closed task. This was also confirmed by the more restrictive statistical test, i.e., *p*-value Kruskal-Wallis < 0.01. The large majority of ML MoS values were positive and higher for patient groups, which can be interpreted as an increase in mechanical stability (MoS) (greater stability of the body configuration) (Curtze et al., 2024; Sangeux et al., 2024). Implying that patients are proactively walking with increased mechanical stability to compensate for a reduced ability to accurately detect and respond to balance disturbances. Although MoS is an instantaneous measure of stability (Sangeux et al., 2024), it cannot easily be used as an indicator of gait stability in a clinical setting. This parameter requires a biomechanical model and calculations, preventing its rapid, and visual assessment. Other studies showed that ML MoS is greater in older people, indicating stabilization attempts to avoid falling (Siragy et al., 2024), or that MoS has the potential to be a useful and objective measure in a variety of clinically affected populations (Watson et al., 2021). Despite the significant results of this study, it would therefore appear that the MoS is not the most appropriate parameter for evaluating patients in a clinical environment. As far as WBAM is concerned, the few differences observed can be explained by the fact that patients’ motor functions are not directly affected, and only the damaged senses result in gait patterns that differ from those of healthy subjects, as already reported in a previous study (Grouvel et al., 2024). This remains to be demonstrated more widely.

Overall, we would like to emphasize that even if parameters seem appropriate to represent dynamic stability, they often seem difficult to calculate and to visually assess in a clinical environment, e.g., WBAM, GaitSD, or MoS, due to complexity of the calculations, and the need for expensive equipment and significant infrastructure. However, the GaitSD parameter seems relevant for discriminating groups and to assess gait variability if more precise analyses are required (Grouvel et al., 2024). For clinical application, we would therefore stress the importance of using parameters that are simple and quick to implement, such as step width, walking speed, CoM displacement, or number of steps.

The clinical applicability of the parameters analyzed was assessed subjectively, on the basis of the researchers’ experience and the feasibility of using the parameters in a clinical environment. The results obtained in this study should be considered as preliminary and should be validated in the future by objective and standardized assessments.

**Figure 4:**
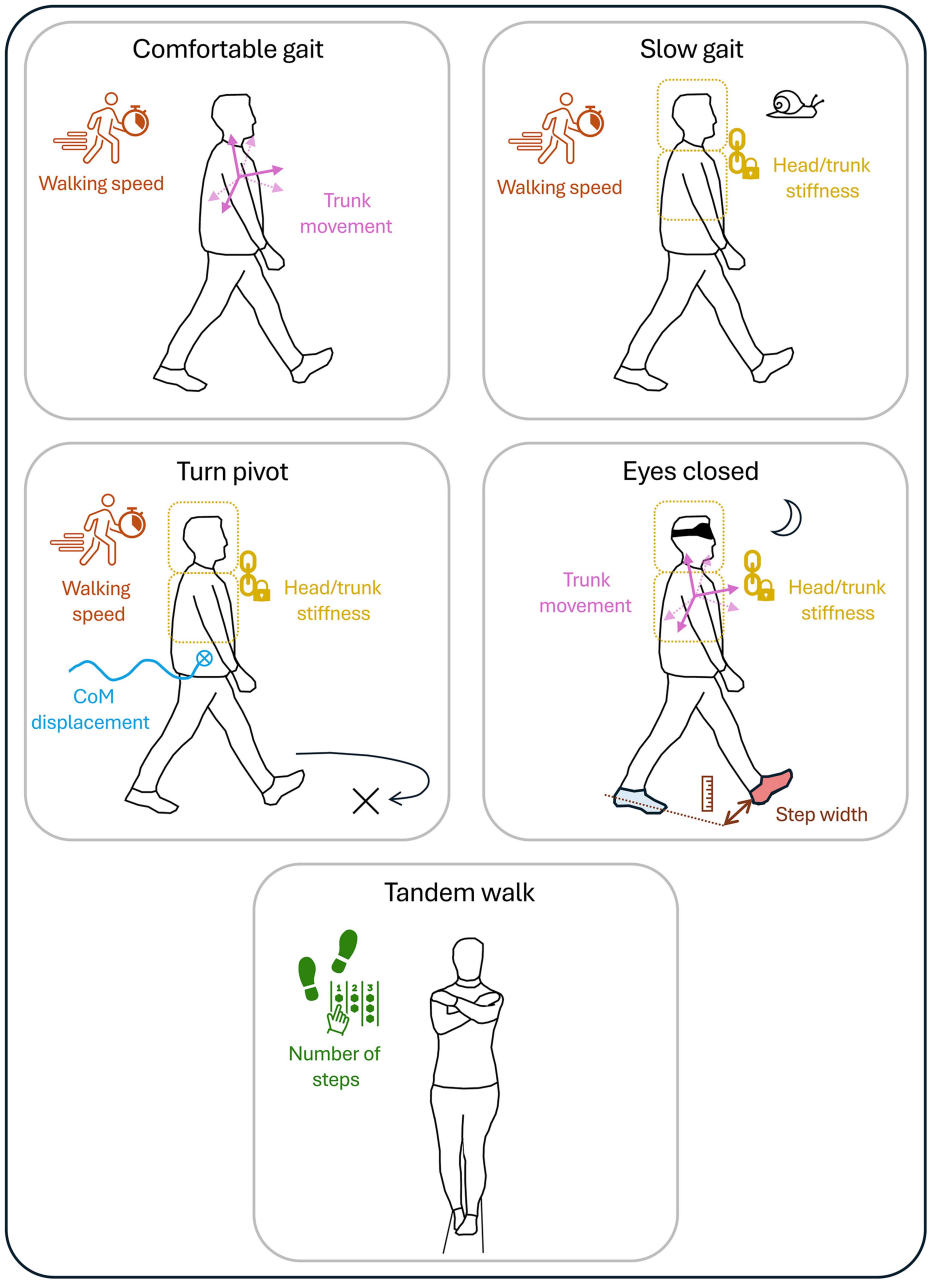
Illustration and summary of all relevant tasks (short-form FGA), and parameters for assessing dynamic stability in patients with bilateral and unilateral vestibulopathy.

Despite the main limitations of this study, in particular the small sample size (due to strict inclusion criteria and limited to the French-speaking part of Switzerland), the heterogeneous compensatory strategies adopted by the patients, the limited number of task repetitions and the absence of control for the participants’ sporting activities or vestibular physiotherapy sessions, these results provide initial indications for clinical practice. Furthermore, a non-parametric calculation of statistical power was performed based on the differences observed in walking speed when participants walked comfortably between the three groups (mean ± sd (median [IQR]): 1.09 ± 0.22 (1.14 [0.19]) m/s for BV; 1.05 ± 0.17 (1.03 [0.12]) m/s for UV; 1.29 ± 0.09 (1.29 [0.15]) m/s for HS). This parameter was chosen as it seems relevant for pathology assessment. The power obtained via a Kruskal-Wallis test was 0.94 (effect size *f* = 0.31; α = 0.05; sample size n = 30; 3 groups). This high power reinforced the sensitivity of the test and the validity of the results of this study. The estimated effect size (f) was moderate. The test was performed with a significance level (α) of 0.05, i.e., a 5% risk of wrongly concluding a difference. The total sample consisted of 30 participants, which, combined with a moderate effect size, explains the high power of the test. These results could help guide the functional follow-up of patients and the evaluation of rehabilitation therapies, such as physiotherapy or vestibular implants, using a simplified testing approach. Table 3 and Figure 4 provide a simplified version of the FGA, highlighting key parameters associated with specific tasks, which could help clinicians to carry out faster and more targeted follow-up assessments of patients. However, these findings should be considered exploratory and interpreted with caution rather than definitive evidence. The parameters that also discriminate with a more restrictive Kruskal Wallis statistical test are shown in Table 3. We can therefore be more certain about these results.

**Table 3:** Relevant set of tasks (short-form FGA), parameters and their interpretation for monitoring the functional status of patients.

Tasks	Parameters to be evaluated by task	Interpretation
Comfortable gait	Walking speed*, trunk movement*	Lower walking speed for patients
Slow gait	**Walking speed**, head/trunk stiffness	Lower walking speed for patients
Turn pivot	**CoM displacement**, **walking speed**, head/trunk stiffness	Higher CoM displacement, lower walking speed for patients
Eyes closed	**Step width**, head/trunk stiffness*, trunk movement	Higher step width, more rigid head/trunk movement for patients
Tandem walk	Number of steps*	Less than 7 steps for patients

Bold: parameters with a good clinical applicability. *Higher degree of certainty for comparison between groups (Kruskal Wallis *p*-value < 0.01).

One important observation concerns the limited differences between BV-UV and UV-HS groups. Indeed, this study also aimed to identify relevant parameters for evaluating rehabilitation therapies such as vestibular implants (Guinand et al., 2015). For the time being, most patients only received a unilateral vestibular implant. In other words, the objective is to bring patients with severe BV to the status of a patient with UV. If no clear difference is found between asymptomatic controls and UV patients, and if differences emerge between BV and the other two groups, this would support the appropriateness of a unilateral implantation strategy. Conversely, if no difference is observed between the BV and UV groups, and if differences appear between UV patients and the asymptomatic group, the efficacy of a unilateral restoration could be questioned. It is also important to highlight the heterogeneity within the UV patient group, with around 30% of patients particularly impacted by their symptoms, a well-documented phenomenon that remains poorly understood (Boutabla et al., 2025; Karabulut et al., 2023).

Finally, although this study identified a promising set of relevant tasks and parameters, the lack of comparable data in the literature restricts direct comparison and interpretability. Further research should aim to fill this gap by exploring additional COSMIN domains and evaluating other psychometric properties of the parameters for each task, including data reliability, responsiveness to treatment and interpretability.

## 5 Conclusion

This study explored dynamic stability in patients with unilateral and bilateral vestibulopathy during multiple motor tasks where compensation from other sensory inputs was limited. The aim was to identify the most relevant parameters and tasks from a set of parameters representing the patients’ dynamic stability. The combination of objective results (discrimination and correlation), together with the observations and expertise of the operators, led to the proposal of a set of 5 tasks: walking at comfortable and slow speeds, walking and turning, walking with eyes closed, and walking on a narrow base. Based on these preliminary results, this study proposes an “short-form FGA” test (SF-FGA), designed to be shorter, clinically practical and better adapted to patients’ symptomatology. This tool could help clinicians visually assess patients’ functional status and monitor rehabilitation therapies. However, these results should be interpreted with caution, as clinical applicability was assessed subjectively, and the sample size was limited. Future studies should focus on validating these results and exploring other psychometric properties of the parameters, as well as assessing patients’ movements in darkness and on uneven ground.

## Data Availability

Raw data in the C3D format are available in Zenodo repositories, doi: 10.5281/zenodo.14236617 for the Comfortable, Slow, and Fast gait tasks; and 10.5281/zenodo.14179651 for the other tasks analyzed in the study, except the Tandem Walk, Backwards, and Steps tasks due to poor quality of data. It is possible to read C3D files with the open-source software Mokka (http://biomechanical-toolkit.github.io/).

## Footnotes

^1^ https://www.c3d.org
