# Supplementary material for: Assessment of dynamic stability and identification of key tasks and parameters in patients with unilateral and bilateral vestibulopathy: a laboratory-based study
Source: Front Neurosci. 2025 Sep 8;19:1624948. doi: 10.3389/fnins.2025.1624948 (PMC12450868; doi:10.3389/fnins.2025.1624948)
Supplement: Supplementary file 1 [file Table_1.docx]

|  | | **Comfortable gait** | | | **Slow gait** | | | **Fast gait** | | | **Double task – Animal** | | | **Double task – Letter** | | |
| --- | --- | --- | --- | --- | --- | --- | --- | --- | --- | --- | --- | --- | --- | --- | --- | --- |
|  |  | **BV** | **UV** | **HS** | **BV** | **UV** | **HS** | **BV** | **UV** | **HS** | **BV** | **UV** | **HS** | **BV** | **UV** | **HS** |
| ***Stability*** | **ML CoM rom (mm)** | 1.08e+02  *(7.64e+01;1.21e+02)* | 7.51e+01 *(6.05e+01;1.03e+02)* | 6.94e+01 *(5.35e+01;7.62e+01)* | 1.07e+02 *(8.12e+01;1.21e+02)* | 8.75e+01 *(8.27e+01;9.76e+01)* | 8.40e+01 *(5.95e+01;9.67e+01)* | 9.32e+01 *(6.00e+01;9.98e+01)* | 5.90e+01 *(5.55e+01;8.84e+01)* | 5.69e+01 *(4.45e+01;7.21e+01)* | 1.11e+02 *(8.81e+01;1.41e+02)* | 9.66e+01 *(7.87e+01;1.24e+02)* | 8.96e+01 *(7.01e+01;1.08e+02)* | 1.23e+02 *(8.13e+01;1.45e+02)* | 1.25e+02 *(1.04e+02;1.42e+02)* | 9.25e+01 *(7.22e+01;1.02e+02)* |
|  |  | η² = 0.167, p = 0.089 | | | η² = 0.123, p = 0.168 | | | η² = 0.125, p = 0.163 | | | η² = 0.065, p = 0.389 | | | η² = 0.132, p = 0.147 | | |
|  | **ML MoS (mm)** | 5.22e+01 *(4.38e+01;5.71e+01)* | 4.77e+01 *(4.45e+01;5.19e+01)* | 4.53e+01 *(4.30e+01;4.65e+01)* | 4.89e+01 *(4.44e+01;5.23e+01)* | 4.75e+01 *(3.74e+01;4.96e+01)* | 4.20e+01 *(3.77e+01;4.69e+01)* | 5.77e+01 *(4.58e+01;6.89e+01)* | 4.55e+01 *(3.95e+01;5.61e+01)* | 5.56e+01 *(4.73e+01;6.28e+01)* | 5.21e+01 *(4.49e+01;5.97e+01)* | 3.86e+01 *(3.41e+01;4.45e+01)* | 4.56e+01 *(4.03e+01;4.80e+01)* | 5.16e+01 *(4.23e+01;5.49e+01)* | 5.09e+01 *(3.80e+01;5.46e+01)* | 4.13e+01 *(3.67e+01;4.63e+01)* |
|  |  | η² = 0.041, p = 0.550 | | | η² = 0.054, p = 0.459 | | | η² = 0.076, p = 0.330 | | | **η² = 0.265, p = 0.021* (BV/UV : p = 0.017*)** | | | η² = 0.063, p = 0.404 | | |
|  | **AP MoS (mm)** | -1.42e+02 *(-1.72e+02;*  *-1.03e+02)* | -1.07e+02 *(-1.30e+02;*  *-8.62e+01)* | -1.68e+02 *(-1.90e+02;*  *-1.59e+02)* | -4.43e+01 *(-7.65e+01;*  *-2.16e+01)* | -2.84e+01 *(-4.40e+01;*  *-1.61e+01)* | -5.99e+01 *(-7.86e+01;*  *-1.22e+01)* | -2.64e+02 *(-3.26e+02;*  *-2.31e+02)* | -2.81e+02 *(-3.19e+02;*  *-2.53e+02)* | -3.27e+02 *(-3.58e+02;*  *-3.03e+02)* | -4.29e+01 *(-1.01e+02;*  *-1.84e+01)* | -3.52e+01 *(-9.12e+01;*  *-3.63e+00)* | -7.67e+01 *(-1.02e+02;*  *-4.48e+01)* | -3.87e+01 *(-6.95e+01;-1.92e-01)* | -2.40e+01 *(-6.79e+01;6.93e+00)* | -4.76e+01 *(-9.03e+01;-8.99e+00)* |
|  |  | **η² = 0.305, p = 0.012* (UV/HS : p = 0.009**)** | | | η² = 0.039, p = 0.572 | | | η² = 0.118, p = 0.181 | | | η² = 0.043, p = 0.538 | | | η² = 0.017, p = 0.777 | | |
|  | **sag WBAM (ND)** | 8.46e-03 *(7.77e-03;9.46e-03)* | 8.33e-03 *(7.75e-03;8.96e-03)* | 8.22e-03 *(7.75e-03;9.44e-03)* | 9.02e-03 *(8.02e-03;9.97e-03)* | 8.12e-03 *(7.43e-03;9.50e-03)* | 8.96e-03 *(7.89e-03;1.01e-02)* | 7.84e-03 *(6.65e-03;9.56e-03)* | 8.24e-03 *(7.11e-03;1.01e-02)* | 7.54e-03 *(6.88e-03;9.45e-03)* | 8.41e-03 *(7.77e-03;9.57e-03)* | 6.70e-03 *(7.37e-03;9.55e-03)* | 8.81e-03 *(7.77e-03;1.05e-02)* | 8.53e-03 *(7.90e-03;9.64e-03)* | 8.73e-03 *(7.83e-03;1.03e-02)* | 8.99e-03 *(7.57e-03;1.04e-02)* |
|  |  | η² < 0.001, p = 0.995 | | | η² = 0.049, p = 0.494 | | | η² = 0.022, p = 0.731 | | | η² = 0.012, p = 0.842 | | | η² = 0.002, p = 0.966 | | |
|  | **fro WBAM (ND)** | 7.71e-03 *(7.37e-03;8.80e-03)* | 8.48e-03 *(8.30e-03;9.33e-03)* | 8.86e-03 *(7.67e-03;1.03e-02)* | 8.32e-03 *(7.60e-03;8.80e-03)* | 8.20e-03 *(7.37e-03;8.87e-03)* | 7.51e-03 *(6.93e-03;9.16e-03)* | 8.40e-03 *(8.11e-03;9.64e-03)* | 9.40e-03 *(8.83e-03;9.91e-03)* | 8.57e-03 *(7.82e-03;9.72e-03)* | 5.99e-03 *(5.31e-03;6.46e-03)* | 6.09e-03 *(4.80e-03;7.23e-03)* | 4.81e-03 *(4.08e-03;6.82e-03)* | 6.21e-03 *(5.42e-03;6.85e-03)* | 6.76e-03 *(5.24e-03;7.71e-03)* | 4.97e-03 *(4.57e-03;6.28e-03)* |
|  |  | η² = 0.094, p = 0.256 | | | η² = 0.014, p = 0.818 | | | η² = 0.097, p = 0.244 | | | η² = 0.029, p = 0.652 | | | η² = 0.066, p = 0.385 | | |
|  | **cor WBAM (ND)** | 5.42e-03 (4.67e-03;5.73e-03) | 4.87e-03 *(4.70e-03;5.27e-03)* | 5.35e-03 *(5.09e-03;5.44e-02)* | 4.29e-03 *(3.93e-03;4.91e-03)* | 4.08e-03 *(3.60e-03;4.64e-03)* | 4.01e-03 *(3.93e-03;4.28e-03)* | 6.67e-03 *(6.34e-03;7.35e-03)* | 6.36e-03 *(5.98e-03;6.51e-03)* | 6.50e-03 *(6.09e-03;6.88e-03)* | 4.47e-03 *(4.21e-03;4.60e-03)* | 4.23e-03 *(4.03e-03;4.36e-03)* | 4.33e-03 *(4.21e-03;4.58e-03)* | 4.41e-03 *(3.66e-03;4.57e-03)* | 3.96e-03 *(3.78e-03;4.21e-03)* | 3.80e-03 *(3.56e-03;4.12e-03)* |
|  |  | η² = 0.088, p = 0.278 | | | η² = 0.023, p = 0.719 | | | η² = 0.053, p = 0.463 | | | η² = 0.056, p = 0.443 | | | η² = 0.066, p = 0.628 | | |
| ***Spatial*** | **CoM score (ND)** | 1.57e+02 *(1.27e+02;2.00e+02)* | 1.35e+02 *(1.00e+02;1.66e+02)* | 1.07e+02 *(1.00e+02;1.31e+02)* | 1.46e+02 *(1.40e+02;1.57e+02)* | 1.23e+02 *(1.15e+02;1.50e+02)* | 1.31e+02 *(1.03e+02;1.46e+02)* | 1.57e+02 *(1.24e+02;1.70e+02)* | 1.14e+02 *(1.00e+02;1.29e+02)* | 1.02e+02 *(1.00e+02;1.53e+02)* | 1.56e+02 *(1.35e+02;1.78e+02)* | 1.52e+02 *(1.19e+02;2.08e+02)* | 1.21e+02 *(1.04e+02;1.59e+02)* | 1.39e+02 *(1.11e+02;2.26e+02)* | 1.31e+02 *(1.17e+02;1.52e+02)* | 1.32e+02 *(1.14e+02;1.96e+02)* |
|  |  | η² = 0.169, p = 0.086 | | | η² = 0.105, p = 0.219 | | | η² = 0.086, p = 0.287 | | | η² = 0.033, p = 0.619 | | | η² = 0.003, p = 0.961 | | |
|  | **Foot score (ND)** | 7.37e+02 *(6.04e+02;9.14e+02)* | 6.97e+02 *(5.38e+02;9.69e+02)* | 5.29e+02 *(4.06e+02;5.90e+02)* | 7.83e+02 *(6.40e+02;9.15e+02)* | 7.19e+02 *(4.74e+02;8.82e+02)* | 5.46e+02 *(4.99e+02;6.02e+02)* | 7.80e+02 *(6.02e+02;8.58e+02)* | 6.25e+02 *(4.89e+02;7.97e+02)* | 6.08e+02 *(4.39e+02;6.97e+02)* | 7.74e+02 *(5.83e+02;8.74e+02)* | 8.12e+02 *(5.82e+02;9.73e+02)* | 5.50e+02 *(4.17e+02;7.77e+02)* | 9.01e+02 *(6.42e+02;1.07e+03)* | 6.31e+02 *(5.56e+02;8.71e+02)* | 6.54e+02 *(5.17e+02;7.60e+02)* |
|  |  | η² = 0.176, p = 0.078 | | | η² = 0.087, p = 0.285 | | | η² = 0.073, p = 0.346 | | | η² = 0.114, p = 0.191 | | | η² = 0.064, p = 0.393 | | |
|  | **Step width (mm)** | 1.02e+02 *(7.53e+01;1.20e+02)* | 8.26e+01 *(7.39e+01;1.17e+02)* | 9.08e+01 *(4.94e+01;9.92e+01)* | 8.99e+01 *(7.79e+01;1.28e+02)* | 7.50e+01 *(6.06e+01;1.05e+02)* | 5.77e+01 *(4.86e+01;7.63e+01)* | 1.06e+02 *(9.92e+01;1.23e+02)* | 7.90e+01 *(6.99e+01;1.08e+02)* | 8.30e+01 *(5.54e+01;9.70e+01)* | 1.28e+02 *(9.61e+01;1.39e+02)* | 8.15e+01 *(5.55e+01;1.06e+02)* | 8.82e+01 *(6.11e+01;1.07e+02)* | 1.24e+02 *(1.02e+02;1.43e+02)* | 8.60e+01 *(6.87e+01;1.21e+02)* | 7.39e+01 *(6.25e+01;1.18e+02)* |
|  |  | η² = 0.034, p = 0.607 | | | η² = 0.190, p = 0.063 | | | η² = 0.111, p = 0.200 | | | η² = 0.082, p = 0.303 | | | η² = 0.161, p = 0.096 | | |
|  | **ML step distance (mm)** | 7.50e+01 *(6.58e+01;9.52e+01)* | 6.53e+01 *(4.50e+01;7.28e+01)* | 5.33e+01 *(4.30e+01;6.65e+01)* | 6.44e+01 *(5.49e+01;8.71e+01)* | 5.29e+01 *(3.31e+01;7.53e+01)* | 4.71e+01 *(3.39e+01;5.57e+01)* | 7.27e+01 *(6.52e+01;9.07e+01)* | 6.23e+01 *(5.42e+01;7.27e+01)* | 5.99e+01 *(4.15e+01;6.33e+01)* | 4.85e+01 *(3.21e+01;6.00e+01)* | 7.18e+01 *(4.81e+01;9.12e+01)* | 5.71e+01 *(4.82e+01;6.71e+01)* | 4.25e+01 *(3.35e+01;5.91e+01)* | 6.89e+01 *(5.76e+01;9.72e+01)* | 4.40e+01 *(2.84e+01;7.06e+01)* |
|  |  | η² = 0.122, p = 0.171 | | | η² = 0.185, p = 0.068 | | | η² = 0.133, p = 0.146 | | | η² = 0.124, p = 0.166 | | | η² = 0.141, p = 0.129 | | |
|  | **Step number (ND)** |  | | |  | | |  | | |  | | |  | | |
| ***Temporal*** | **Walking speed (m/s)** | 1.14e+00 *(1.04e+00;1.23e+00)* | 1.03e+00 *(9.87e-01;1.11e+00)* | 1.29e+00 *(1.21e+00;1.36e+00)* | 7.71e-01 *(7.49e-01;8.93e-01)* | 7.21e-01 *(6.92e-01;7.43e-01)* | 9.15e-01 *(8.62e-01;9.61e-01)* | 1.54e+00 *(1.44e+00;1.75e+00)* | 1.55e+00 *(1.44e+00;1.69e+00)* | 1.78e+00 *(1.75e+00;1.83e+00)* | 8.05e-01 *(5.82e-01;9.09e-01)* | 8.31e-01 *(7.34e-01;8.70e-01)* | 9.81e-01 *(8.73e-01;1.10e+00)* | 6.77e-01 *(5.87e-01;8.27e-01)* | 6.61e-01 *(5.80e-01;7.69e-01)* | 8.94e-01 *(7.70e-01;1.01e+00)* |
|  |  | ♦ **η² = 0.345, p = 0.007** (UV/HS : p = 0.006**)** | | | **η² = 0.296, p = 0.014* (UV/HS : p = 0.010*)** | | | **η² = 0.212, p = 0.047* -** | | | η² = 0.143, p = 0.125 | | | **η² = 0.249, p = 0.027* (BV/HS : p = 0.048*)** | | |
|  | **Double support time (s)** | 2.45e-01 *(2.29e-01;2.69e-01)* | 3.03e-01 *(2.68e-01;3.28e-01)* | 2.55e-01 *(2.23e-01;2.64e-01)* | 3.53e-01 *(3.50e-01;3.94e-01)* | 4.10e-01 *(3.91e-01;4.79e-01)* | 3.40e-01 *(3.23e-01;4.24e-01)* | 1.70e-01 *(1.50e-01;2.20e-01)* | 1.83e-01 *(1.73e-01;1.93e-01)* | 1.80e-01 *(1.66e-01;1.80e-01)* | 3.40e-01 *(2.85e-01;3.60e-01)* | 3.70e-01 *(3.04e-01;3.99e-01)* | 3.40e-01 *(2.86e-01;3.44e-01)* | 3.73e-01 *(3.35e-01;4.23e-01)* | 4.65e-01 *(3.66e-01;4.80e-01)* | 3.70e-01 *(3.30e-01;4.34e-01)* |
|  |  | **η² = 0.234, p = 0.034* (UV/HS : p = 0.047*)** | | | η² = 0.199, p = 0.056 | | | η² = 0.036, p = 0.595 | | | η² = 0.061, p = 0.412 | | | η² = 0.060, p = 0.418 | | |
|  | **Task time (s)** |  | | |  | | |  | | |  | | |  | | |
| ***Kinematic*** | **Head AI – Roll (ND)** | -1.02e-01 *(-1.32e-01;-1.15e-02)* | -8.38e-02 *(-1.54e-01;1.59e-02)* | -1.78e-02 *(-8.39e-02;1.93e-02)* | -1.04e-01 *(-1.83e-01;-1.23e-02)* | -2.26e-01 *(-2.69e-01;-1.35e-01)* | -5.80e-03 *(-6.12e-02;6.55e-02)* | -1.10e-01 *(-3.57e-01;-1.37e-02)* | -8.31e-02 *(-2.30e-01;4.32e-03)* | 3.69e-02 *(-1.02e-01;1.61e-01)* | -8.85e-02 *(-2.56e-01;-2.62e-02)* | -4.02e-02 *(-8.86e-02;1.80e-02)* | 6.06e-02 *(-3.19e-02;2.12e-01)* | -2.19e-02 *(-6.55e-02;6.38e-02)* | -1.42e-01 *(-2.16e-01;-3.61e-02)* | 5.77e-02 *(-1.06e-01;1.94e-01)* |
|  |  | η² = 0.061, p = 0.414 | | | **η² = 0.223, p = 0.039* (UV/HS : p = 0.035*)** | | | η² = 0.080, p = 0.174 | | | η² = 0.126, p = 0.161 | | | η² = 0.114, p = 0.191 | | |
|  | **Head AI – Pitch (ND)** | -7.79e-02 *(-2.97e-01;2.50e-01)* | -1.66e-01 *(-3.55e-01;4.76e-02)* | 1.85e-01 *(-3.42e-01;3.98e-01)* | -3.77e-02 *(-4.03e-01;2.23e-01)* | -2.53e-01 *(-4.17e-01;3.12e-02)* | -1.48e-01 *(-3.82e-01;1.31e-01)* | 4.83e-02 *(-7.21e-02;1.34e-01)* | -1.67e-02 *(-1.64e-01;1.04e-01)* | 2.17e-01 *(-2.74e-01;3.26e-01)* | -8.39e-02 *(-3.85e-01;1.20e-01)* | -3.20e-01 *(-3.84e-01;-8.36e-02)* | -4.01e-01 *(-6.08e-01;-2.06e-01)* | -3.92e-02 *(-5.09e-01;2.27e-01)* | -4.16e-01 *(-4.87e-01;-3.09e-02)* | -1.68e-01 *(-5.25e-01;-1.10e-01)* |
|  |  | η² = 0.051, p = 0.476 | | | η² = 0.020, p = 0.744 | | | η² = 0.052, p = 0.468 | | | η² = 0.086, p = 0.288 | | | η² = 0.032, p = 0.625 | | |
|  | **Head AI – Yaw (ND)** | 9.53e-02 *(-1.32e-01;3.58e-01)* | 3.39e-01 *(-9.97e-02;4.27e-01)* | 2.28e-01 *(1.31e-01;4.38e-01)* | -2.30e-01 *(-4.22e-01;6.24e-03)* | 1.44e-01 *(1.72e-02;2.59e-01)* | 9.23e-02 *(4.84e-02;1.42e-01)* | 2.03e-01 *(-1.46e-01;4.81e-01)* | 5.10e-01 *(3.53e-01;7.04e-01)* | 6.13e-01 *(4.75e-01;6.55e-01)* | -1.62e-01 *(-4.06e-01;1.04e-01)* | 1.48e-01 *(7.30e-03;2.63e-01)* | 2.23e-01 *(-4.68e-02;5.25e-01)* | -1.60e-01 *(-3.16e-01;1.96e-01)* | 2.00e-01 *(4.30e-02;4.26e-01)* | -3.10e-02 *(-8.49e-02;6.75e-02)* |
|  |  | η² = 0.036, p = 0.598 | | | **η² = 0.209, p = 0.048* -** | | | **η² = 0.258, p = 0.024* (BV/HS : p = 0.025*)** | | | η² = 0.161, p = 0.096 | | | η² = 0.094, p = 0.256 | | |
|  | **Head AV (rad/s)** | 2.42e-01 *(2.04e-01;2.71e-01)* | 3.13e-01 *(2.25e-01;3.49e-01)* | 3.11e-01 *(2.23e-01;3.33e-01)* | 2.00e-01 *(1.92e-01;2.70e-01)* | 2.44e-01 *(2.10e-01;3.32e-01)* | 2.23e-01 *(2.08e-01;2.83e-01)* | 2.79e-01 *(2.48e-01;3.24e-01)* | 3.25e-01 *(2.84e-01;4.24e-01)* | 2.97e-01 *(2.75e-01;3.93e-01)* | 3.03e-01 *(2.48e-01;3.18e-01)* | 3.67e-01 *(2.64e-01;5.16e-01)* | 4.03e-01 *(2.67e-01;4.68e-01)* | 2.30e-01 *(2.08e-01;3.55e-01)* | 3.71e-01 *(2.84e-01;7.37e-01)* | 3.38e-01 *(2.40e-01;6.02e-01)* |
|  |  | η² = 0.094, p = 0.257 | | | η² = 0.100, p = 0.235 | | | η² = 0.068, p = 0.374 | | | η² = 0.112, p = 0.192 | | | η² = 0.154, p = 0.107 | | |
|  | **Trunk AV (rad/s)** | 1.24e+00 *(1.08e+00;1.37e+00)* | 1.21e+00 *(1.15e+00;1.47e+00)* | 1.57e+00 *(1.56e+00;1.63e+00)* | 9.26e-01 *(8.66e-01;1.02e+00)* | 8.65e-01 *(8.02e-01;1.00e-00)* | 1.11e+00 *(9.07e-01;1.14e+00)* | 1.69e+00 *(1.50e+00;1.82e+00)* | 1.71e+00 *(1.64e+00;1.83e+00)* | 1.94e+00 *(1.88e+00;2.05e+00)* | 8.68e-01 *(6.70e-01;1.08e+00)* | 9.05e-01 *(7.86e-01;1.02e+00)* | 1.03e+00 *(9.76e-01;1.05e+00)* | 8.11e-01 *(7.06e-01;8.78e-01)* | 8.97e-01 *(7.27e-01;1.02e+00)* | 1.05e+00 *(9.22e-01;1.12e+00)* |
|  |  | ♦ **η² = 0.438, p = 0.002* (BV/HS : p = 0.005**; UV/HS : p = 0.005**)** | | | η² = 0.165, p = 0.092 | | | **η² = 0.235, p = 0.033* (BV/HS : p = 0.038*)** | | | η² = 0.144, p = 0.122 | | | **η² = 0.229, p = 0.036* (BV/HS : p = 0.033*)** | | |
|  | **GaitSD (°)** | 1.82e+00 *(1.57e+00;1.99e+00)* | 1.40e+00 *(1.30e+00;1.51e+00)* | 1.38e+00 *(1.27e+00;1.55e+00)* | 1.84e+00 *(1.62e+00;2.40e+00)* | 1.69e+00 *(1.46e+00;1.92e+00)* | 1.51e+00 *(1.32e+00;1.77e+00)* | 1.97e+00 *(1.86e+00;2.40e+00)* | 1.44e+00 *(1.26e+00;1.79e+00)* | 1.54e+00 *(1.41e+00;1.68e+00)* | 2.28e+00 *(2.08e+00;2.79e+00)* | 1.76e+00 *(1.38e+00;1.92e+00)* | 1.53e+00 *(1.34e+00;1.79e+00)* | 2.16e+00 *(1.95e+00;2.66e+00)* | 2.11e+00 *(1.83e+00;2.28e+00)* | 1.71e+00 *(1.36e+00;1.92e+00)* |
|  |  | η² = 0.148, p = 0.117 | | | η² = 0.126, p = 0.161 | | | ♦ **η² = 0.368, p = 0.005** (BV/HS : p = 0.007**; BV/UV : p = 0.019*)** | | | **η² = 0.292, p = 0.014* (BV/HS : p = 0.027*; BV/UV : p = 0.032*)** | | | η² = 0.128, p = 0.155 | | |

Supplementary Table 1- A. Median results (Q1;Q3) for all parameters calculated for each task, and results of Kruskal-Wallis test (alpha = 0.05), and in case of significant result, result of Dunn's post hoc test with Holm correction. *p ≤ 0.05; **p ≤ 0.01; ***p ≤ 0.001. Data for which the Kruskal-Wallis test, with alpha set at 0.01, is significant, are indicated by a ♦.

|  | | **Change speed** | | | **Horizontal head turns** | | | **Vertical head turns** | | | **Turn pivot** | | | **Step obstacle** | | |
| --- | --- | --- | --- | --- | --- | --- | --- | --- | --- | --- | --- | --- | --- | --- | --- | --- |
|  |  | **BV** | **UV** | **HS** | **BV** | **UV** | **HS** | **BV** | **UV** | **HS** | **BV** | **UV** | **HS** | **BV** | **UV** | **HS** |
| ***Stability*** | **ML CoM rom (mm)** | 1.03e+02 *(7.28e+01;1.48e+02)* | 1.03e+02 *(9.41e+01;1.29e+02)* | 7.50e+01 *(6.79e+01;8.78e+01)* | 1.28e+02 *(1.09e+02;1.44e+02)* | 1.24e+02 *(1.05e+02;1.79e+02)* | 9.55e+01 *(8.66e+01;9.85e+01)* | 1.17e+02 *(1.03e+02;1.38e+02)* | 9.25e+01 *(7.82e+01;1.20e+02)* | 8.28e+01 *(6.36e+01;1.02e+02)* | 1.31e+02 *(9.02e+01;2.14e+02)* | 1.18e+02 *(1.02e+02;1.34e+02)* | 8.91e+01 *(6.78e+01;1.12e+02)* | 9.51e+01 *(8.14e+01;1.13e+02)* | 1.06e+02 *(5.93e+01;1.18e+02)* | 6.30e+01 *(5.40e+01;7.24e+01)* |
|  |  | η² = 0.180. p = 0.074 | | | η² = 0.195, p = 0.059 | | | **η² = 0.279, p = 0.018* (BV/HS : p = 0.016*)** | | | η² = 0.147, p = 0.119 | | | η² = 0.198, p = 0.057 | | |
|  | **ML MoS (mm)** | 5.31e+01 *(4.43e+01;6.06e+01)* | 4.77e+01 *(4.50e+01;5.41e+01)* | 4.44e+01 *(3.48e+01;5.09e+01)* | 5.63e+01 *(5.12e+01;7.51e+01)* | 5.45e+01 *(4.20e+01;5.72e+01)* | 4.94e+01 *(4.54e+01;5.74e+01)* | 5.33e+01 *(4.71e+01;6.17e+01)* | 5.03e+01 *(4.14e+01;5.95e+01)* | 4.15e+01 *(3.71e+01;5.13e+01)* | 4.31e+01 *(3.93e+01;6.36e+01)* | 3.52e+01 *(1.81e+01;4.61e+01)* | 4.24e+01 *(3.77e+01;5.20e+01)* | 4.76e+01 *(4.26e+01;5.08e+01)* | 4.46e+01 *(4.13e+01;4.72e+01)* | 4.15e+01 *(3.40e+01;4.43e+01)* |
|  |  | η² = 0.045, p = 0.520 | | | η² = 0.052, p = 0.468 | | | η² = 0.103, p = 0.224 | | | η² = 0.090, p = 0.272 | | | η² = 0.049, p = 0.490 | | |
|  | **AP MoS (mm)** | -1.45e+02 *(-1.60e+02;*  *-1.34e+02)* | -1.27e+02 *(-2.15e+02;*  *-1.11e+02)* | -1.65e+02 *(-1.87e+02;*  *-1.33e+02)* | -6.75e+01 *(-7.92e+01;*  *-3.80e+01)* | -7.49e+01 *(-8.35e+01;*  *-5.87e+01)* | -8.45e+01 *(-9.75e+01;*  *-6.68e+01)* | -8.18e+01 *(-9.12e+01;*  *-6.82e+01)* | -8.06e+01 *(-9.94e+01;*  *-5.82e+01)* | -1.12e+02 *(-1.28e+02;*  *-8.83e+01)* | -8.18e+01 *(-1.36e+02;5.07e+01)* | -4.81e+01 *(-1.03e+02;*  *-1.80e+01)* | -6.49e+00 *(-7.41e+01;7.21e+00)* | -9.66e+01 *(-1.48e+02;*  *-4.98e+01)* | -1.03e+02 *(-1.25e+02;*  *-6.73e+01)* | -9.11e+01 *(-1.35e+02;-7.43e+01)* |
|  |  | η² = 0.010, p = 0.860 | | | η² = 0.043, p = 0.533 | | | **η² = 0.227, p = 0.037* -** | | | η² = 0.033, p = 0.619 | | | η² = 0.012, p = 0.842 | | |
|  | **sag WBAM (ND)** | 8.00e-03 *(6.76e-03;9.18e-03)* | 8.16e-03 *(7.65e-03;8.43e-03)* | 8.45e-03 *(7.22e-03;1.09e-02)* | 7.47e-03 *(6.27e-03;8.49e-03)* | 7.72e-03 *(6.49e-03;8.72e-03)* | 7.55e-03 *(7.01e-03;7.80e-03)* | 8.19e-03 *(6.95e-03;9.53e-03)* | 8.28e-03 *(6.70e-03;8.62e-03)* | 7.75e-03 *(7.36e-03;7.95e-03)* |  | | |  | | |
|  |  | η² = 0.016, p = 0.790 | | | η² = 0.009, p = 0.882 | | | η² = 0.009, p = 0.879 | | |  |  |  |  |  |  |
|  | **fro WBAM (ND)** | 7.75e-03 *(6.15e-03;8.77e-03)* | 8.03e-03 *(7.26e-03;8.87e-03)* | 8.18e-03 *(6.81e-03;1.01e-02)* | 6.72e-03 *(6.01e-03;7.26e-03)* | 7.84e-03 *(6.40e-03;8.46e-03)* | 7.09e-03 *(6.23e-03;7.45e-03)* | 7.30e-03 *(6.61e-03;8.58e-03)* | 7.98e-03 *(6.71e-03;8.41e-03)* | 7.72e-03 *(6.93e-03;8.17e-03)* |  | | |  | | |
|  |  | η² = 0.043, p = 0.538 | | | η² = 0.074, p = 0.342 | | | η² = 0.009, p = 0.882 | | |  |  |  |  |  |  |
|  | **cor WBAM (ND)** | 5.35e-03 *(4.42e-03;6.04e-03)* | 5.50e-03 *(5.13e-03;5.79e-03)* | 5.35e-03 *(5.01e-03;5.47e-03)* | 4.51e-03 *(3.86e-03;4.74e-03)* | 4.36e-03 *(4.09e-03;4.48e-03)* | 4.14e-03 *(3.91e-03;4.93e-03)* | 4.45e-03 *(3.87e-03;5.01e-03)* | 4.34e-03 *(4.20e-03;4.63e-03)* | 4.25e-03 *(4.08e-03;4.99e-03)* |  | | |  | | |
|  |  | η² = 0.023, p = 0.716 | | | η² < 0.001, p = 0.988 | | | η² < 0.001, p = 0.995 | | |  |  |  |  |  |  |
| ***Spatial*** | **CoM score (ND)** | 1.52e+02 *(1.01e+02;1.97e+02)* | 1.80e+02 *(1.26e+02;2.52e+02)* | 1.04e+02 *(1.00e+02;1.35e+02)* | 1.47e+02 *(1.23e+02;2.14e+02)* | 1.89e+02 *(1.42e+02;3.29e+02)* | 1.18e+02 *(1.08e+02;1.30e+02)* | 1.40e+02 *(1.20e+02;2.65e+02)* | 1.23e+02 *(1.00e+02;1.49e+02)* | 1.28e+02 *(1.09e+02;1.38e+02)* | 2.48e+02 *(1.40e+02;3.36e+02)* | 1.46e+02 *(1.38e+02;1.95e+02)* | 1.26e+02 *(1.00e+02;1.45e+02)* | 1.22e+02 *(1.02e+02;1.56e+02)* | 1.65e+02 *(1.21e+02;2.12e+02)* | 1.00e+02 *(1.00e+02;1.28e+02)* |
|  |  | η² = 0.179, p = 0.075 | | | **η² = 0.242, p = 0.030* (UV/HS : p = 0.031*)** | | | η² = 0.092, p = 0.263 | | | **η² = 0.207, p = 0.050* (BV/HS : p = 0.044*)** | | | η² = 0.148, p = 0.117 | | |
|  | **Foot score (ND)** | 7.27e+02 *(6.19e+02;9.63e+02)* | 7.68e+02 *(6.80e+02;8.32e+02)* | 4.28e+02 *(4.00e+02;5.03e+02)* | 7.48e+02 *(6.60e+02;1.02e+03)* | 8.79e+02 *(7.04e+02;1.02e+03)* | 4.90e+02 *(4.51e+02;6.64e+02)* | 7.59e+02 *(6.08e+02;9.29e+02)* | 6.70e+02 *(5.91e+02;8.99e+02)* | 5.44e+02 *(4.46e+02;6.58e+02)* | 9.46e+02 *(6.70e+02;1.19e+03)* | 8.54e+02 *(7.14e+02;9.98e+02)* | 7.32e+02 *(4.75e+02;8.41e+02)* | 7.28e+02 *(5.59e+02;8.85e+02)* | 8.89e+02 *(5.44e+02;9.57e+02)* | 4.56e+02 *(3.37e+02;7.84e+02)* |
|  |  | η² = 0.199, p = 0.056 | | | **η² = 0.242, p = 0.030* -** | | | η² = 0.141, p = 0.129 | | | η² = 0.110, p = 0.203 | | | η² = 0.192, p = 0.061 | | |
|  | **Step width (mm)** | 9.47e+01 *(8.67e+01;1.15e+02)* | 9.02e+01 *(5.89e+01;1.13e+02)* | 8.07e+01 *(3.98e+01;1.13e+02)* | 1.16e+02 *(9.23e+01;1.36e+02)* | 9.04e+01 *(7.33e+01;9.62e+01)* | 6.43e+01 *(4.73e+01;1.03e+02)* | 1.07e+02 *(7.60e+01;1.48e+02)* | 9.11e+01 *(8.05e+01;1.11e+02)* | 7.35e+01 *(4.50e+01;9.07e+01)* | 1.01e+02 *(7.87e+01;1.27e+02)* | 7.71e+01 *(5.24e+01;1.15e+02)* | 7.55e+01 *(3.87e+01;9.84e+01)* | 9.92e+01 *(7.78e+01;1.13e+02)* | 8.04e+01 *(5.61e+01;1.19e+02)* | 5.95e+01 *(3.30e+01;1.11e+02)* |
|  |  | η² = 0.039, p = 0.568 | | | η² = 0.173, p = 0.082 | | | η² = 0.131, p = 0.149 | | | η² = 0.050, p = 0.481 | | | η² = 0.056, p = 0.445 | | |
|  | **ML step distance (mm)** | 8.76e+01 *(5.74e+01;1.09e+02)* | 6.90e+01 *(6.14e+01;8.66e+01)* | 5.72e+01 *(5.15e+01;7.12e+01)* | 6.84e+01 *(6.15e+01;1.02e+02)* | 1.04e+02 *(1.00e+02;1.18e+02)* | 7.36e+01 *(5.67e+01;8.01e+01)* | 6.61e+01 *(5.13e+01;8.81e+01)* | 7.77e+01 *(5.53e+01;1.09e+02)* | 5.07e+01 *(3.39e+01;7.27e+01)* | 1.61e+02 *(1.14e+02;2.21e+02)* | 1.88e+02 *(1.74e+02;2.26e+02)* | 1.78e+02 *(7.64e+01;1.98e+02)* | 7.83e+01 *(6.83e+01;1.10e+02)* | 6.00e+01 *(5.23e+01;9.52e+01)* | 4.47e+01 *(3.72e+01;5.91e+01)* |
|  |  | η² = 0.141, p = 0.129 | | | η² = 0.116, p = 0.186 | | | η² = 0.130, p = 0.152 | | | η² = 0.075, p = 0.335 | | | **η² = 0.212, p = 0.046* (BV/HS : p = 0.044*)** | | |
|  | **Step number (ND)** |  | | |  | | |  | | |  | | |  | | |
| ***Temporal*** | **Walking speed (m/s)** | 1.10e+00 *(9.24e-01;1.20e+00)* | 1.13e+00 *(9.17e-01;1.44e+00)* | 1.30e+00 *(1.16e+00;1.36e+00)* | 7.31e-01 *(6.02e-01;9.03e-01)* | 7.91e-01 *(7.65e-01;9.41e-01)* | 9.37e-01 *(8.30e-01;1.01e+00)* | 7.85e-01 *(6.58e-01;9.10e-01)* | 7.99e-01 *(7.45e-01;9.99e-01)* | 1.02e+00 *(9.52e-01;1.09e+00)* | 9.56e-01 *(8.78e-01;1.09e+00)* | 1.01e+00 *(8.97e-01;1.17e+00)* | 1.16e+00 *(1.07e+00;1.28e+00)* | 8.16e-01 *(6.68e-01;1.04e+00)* | 8.96e-01 *(7.31e-01;1.06e+00)* | 1.07e+00 *(9.01e-01;1.10e+00)* |
|  |  | η² = 0.080, p = 0.312 | | | η² = 0.202, p = 0.053 | | | **η² = 0.215, p = 0.045* (BV/HS : p = 0.044*)** | | | **η² = 0.215, p = 0.044* (BV/HS : p = 0.041*)** | | | η² = 0.130, p = 0.153 | | |
|  | **Double support time (s)** | 2.40e-01 *(2.14e-01;2.74e-01)* | 1.90e-01 *(1.70e-01;2.65e-01)* | 2.20e-01 *(1.81e-01;2.71e-01)* | 3.15e-01 *(2.85e-01;3.40e-01)* | 3.40e-01 *(3.15e-01;3.69e-01)* | 3.25e-01 *(3.01e-01;3.54e-01)* | 3.03e-01 *(2.68e-01;3.34e-01)* | 3.38e-01 *(3.26e-01;3.80e-01)* | 2.92e-01 *(2.70e-01;3.29e-01)* | 2.35e-01 *(2.03e-01;2.74e-01)* | 2.93e-01 *(2.70e-01;3.23e-01)* | 2.60e-01 *(2.41e-01;2.73e-01)* | 2.52e-01 *(2.23e-01;2.84e-01)* | 2.70e-01 *(2.43e-01;3.10e-01)* | 2.40e-01 *(2.23e-01;2.70e-01)* |
|  |  | η² = 0.042, p = 0.542 | | | η² = 0.042, p = 0.541 | | | η² = 0.106, p = 0.215 | | | η² = 0.192, p = 0.062 | | | η² = 0.073, p = 0.348 | | |
|  | **Task time (s)** |  | | |  | | |  | | | 1.45e+00 *(1.37e+00;1.54e+00)* | 1.62e+00 *(1.40e+00;*  *1.87e+00)* | 1.29e+00 *(1.13e+00;*  *1.50e+00)* | 1.88e+00 *(1.51e+00;2.41e+00)* | 1.89e+00 *(1.59e+00;*  *2.40e+00)* | 1.56e+00 *(1.43e+00;*  *1.69e+00)* |
|  |  |  |  |  |  |  |  |  |  |  | η² = 0.135, p = 0.140 | | | η² = 0.144, p = 0.125 | | |
| ***Kinematic*** | **Head AI – Roll (ND)** | 1.51e-01 *(-8.94e-02;2.98e-01)* | 9.96e-02 *(-4.83e-02;1.54e-01)* | 9.58e-02 *(3.37e-02;1.78e-01)* | -1.66e-01 *(-4.51e-01;-1.18e-01)* | -2.15e-01 *(-3.58e-01;-1.01e-01)* | 1.29e-01 *(-2.13e-01;3.78e-01)* | -1.57e-01 *(-1.94e-01;-1.27e-01)* | -1.41e-01 *(-1.89e-01;-9.33e-02)* | -8.52e-02 *(-1.14e-01;-3.93e-02)* | -7.61e-01 *(-8.53e-01;-6.53e-01)* | -5.87e-01 *(-6.92e-01;-3.52e-01)* | -5.08e-01 *(-7.98e-01;1.06e-01)* | 9.71e-02 *(-2.86e-01;1.35e-01)* | -3.17e-02 *(-1.98e-01;2.34e-01)* | -2.87e-02 *(-2.02e-01;5.35e-02)* |
|  |  | η² = 0.007, p = 0.907 | | | η² = 0.118, p = 0.180 | | | **η² = 0.306, p = 0.012* (BV/HS : p = 0.013*)** | | | η² = 0.143, p = 0.126 | | | η² = 0.002, p = 0.973 | | |
|  | **Head AI – Pitch (ND)** | 1.34e-01 *(-9.69e-02;2.11e-01)* | -7.24e-02 *(-1.81e-01;-2.30e-02)* | -1.84e-01 *(-3.11e-01;1.09e-01)* | -2.12e-01 *(-5.23e-01;3.01e-01)* | -1.59e-01 *(-3.30e-01;2.14e-01)* | 7.32e-02 *(-6.89e-01;3.50e-01)* | 3.54e-01 *(-1.73e-01;6.74e-01)* | 2.06e-01 *(-4.25e-03;6.02e-01)* | 3.35e-01 *(1.51e-01;5.39e-01)* | -7.74e-01 *(-9.32e-01;-5.84e-01)* | -3.84e-01 *(-4.81e-01;-6.25e-02)* | -6.15e-01 *(-7.33e-01;-4.92e-01)* | 2.25e-01 *(-2.89e-01;3.87e-01)* | -1.19e-02 *(-2.95e-01;1.49e-01)* | -2.91e-01 *(-4.65e-01;4.54e-02)* |
|  |  | η² = 0.047, p = 0.509 | | | η² < 0.001, p = 0.991 | | | η² = 0.016, p = 0.792 | | | **η² = 0.264, p = 0.022* (BV/UV : p = 0.017*)** | | | η² = 0.126, p = 0.162 | | |
|  | **Head AI – Yaw (ND)** | 3.13e-02 *(-3.19e-01;3.58e-01)* | 3.58e-01 *(1.96e-02;5.31e-01)* | 2.79e-01 *(2.07e-01;4.25e-01)* | -2.39e-01 (-2.80e-01;-2.13e-01) | -1.55e-01 (-2.96e-01;-1.27e-01) | -1.22e-01 (-1.40e-01;-8.74e-02) | -7.85e-02 *(-1.55e-01;3.27e-02)* | 2.22e-02 *(-2.70e-01;1.83e-01)* | 2.96e-01 *(1.55e-02;4.49e-01)* | -9.84e-01 *(-9.92e-01;-9.78e-01)* | -9.71e-01 *(-9.85e-01;-9.53e-01)* | -9.84e-01 *(-9.91e-01;-9.67e-01)* | 1.47e-01 *(-1.92e-01;3.52e-01)* | 3.92e-01 *(1.74e-01;4.35e-01)* | 6.08e-01 *(4.16e-01;6.89e-01)* |
|  |  | η² = 0.107, p = 0.213 | | | ♦ **η² = 0.462, p = 0.001** (BV/HS : p < 0.001***)** | | | η² = 0.186, p = 0.067 | | | η² = 0.060, p = 0.420 | | | **η² = 0.232, p = 0.035* (BV/HS : p = 0.029*)** | | |
|  | **Head AV (rad/s)** | 2.65e-01 *(2.28e-01;3.05e-01)* | 2.71e-01 *(2.49e-01;3.30e-01)* | 3.20e-01 *(2.39e-01;3.68e-01)* | 1.26e+00 *(1.08e+00;1.44e+00)* | 1.39e+00 *(1.25e+00;1.52e+00)* | 1.43e+00 *(1.38e+00;1.51e+00)* | 7.40e-01 *(6.58e-01;9.70e-01)* | 1.03e+00 *(9.29e-01;1.23e+00)* | 9.29e-01 *(8.90e-01;1.16e+00)* | 2.96e+01 *(2.50e+01;3.64e+01)* | 3.17e+01 *(2.99e+01;3.77e+01)* | 2.38e+01 *(1.84e+00;2.80e+01)* | 2.69e-01 *(2.41e-01;3.38e-01)* | 3.72e-01 *(3.20e-01;4.46e-01)* | 3.24e-01 *(3.06e-01;3.66e-01)* |
|  |  | η² = 0.058, p = 0.431 | | | η² = 0.073, p = 0.348 | | | **η² = 0.207, p = 0.050* -** | | | η² = 0.144, p = 0.123 | | | η² = 0.147, p = 0.119 | | |
|  | **Trunk AV (rad/s)** | 1.34e+00 *(1.05e+00;1.40e+00)* | 1.41e+00 *(1.21e+00;1.60e+00)* | 1.66e+00 *(1.12e+00;1.84e+00)* | 7.91e-01 *(7.43e-01;1.01e+00)* | 9.44e-01 *(7.68e-01;1.11e+00)* | 9.46e-01 *(8.85e-01;1.06e+00)* | 9.05e-01 *(7.99e-01;9.46e-01)* | 8.83e-01 *(8.29e-01;9.88e-01)* | 1.30e+00 *(9.67e-01;1.33e+00)* | 2.13e+00 *(9.92e-01;2.89e+01)* | 2.88e+01 *(2.40e+01;2.98e+01)* | 1.25e+01 *(1.47e+00;2.61e+01)* | 1.21e+00 *(9.10e-01;1.42e+00)* | 1.14e+00 *(1.10e+00;1.25e+00)* | 1.41e+00 *(1.17e+00;1.56e+00)* |
|  |  | η² = 0.044, p = 0.527 | | | η² = 0.047, p = 0.508 | | | η² = 0.175, p = 0.079 | | | η² = 0.197, p = 0.058 | | | η² = 0.112, p = 0.196 | | |
|  | **GaitSD (°)** | 3.89e+00 *(3.34e+00;4.61e+00)* | 5.06e+00 *(3.52e+00;6.25e+00)* | 3.84e+00 *(3.39e+00;5.52e+00)* | 3.48e+00 *(3.05e+00;5.10e+00)* | 3.00e+00 *(2.57e+00;4.24e+00)* | 2.35e+00 *(2.04e+00;3.26e+00)* | 3.00e+00 *(2.46e+00;3.17e+00)* | 2.28e+00 *(2.21e+00;2.44e+00)* | 1.64e+00 *(1.45e+00;1.86e+00)* | 1.96e+00 *(1.90e+00;3.04e+00)* | 1.39e+00 *(1.25e+00;2.13e+00)* | 2.14e+00 *(2.09e+00;2.70e+00)* | 9.29e+00 *(8.41e+00;9.79e+00)* | 7.25e+00 *(6.81e+00;9.89e+00)* | 8.14e+00 *(6.41e+00;9.25e+00)* |
|  |  | η² = 0.054, p = 0.456 | | | **η² = 0.241, p = 0.030* (BV/HS : p = 0.025*)** | | | ♦ **η² = 0.505, p < 0.001*** (BV/HS : p < 0.001***; UV/HS : p = 0.034*)** | | | η² = 0.077, p = 0.327 | | | η² = 0.069, p = 0.368 | | |

Supplementary Table 1- B. Median results (Q1;Q3) for all parameters calculated for each task, and results of Kruskal-Wallis test (alpha = 0.05), and in case of significant result, result of Dunn's post hoc test with Holm correction. *p ≤ 0.05; **p ≤ 0.01; ***p ≤ 0.001. Data for which the Kruskal-Wallis test, with alpha set at 0.01, is significant, are indicated by a ♦.

|  | | **Eyes closed** | | | **Tandem walk** | | | **Backwards** | | | **Steps** | | |
| --- | --- | --- | --- | --- | --- | --- | --- | --- | --- | --- | --- | --- | --- |
|  |  | **BV** | **UV** | **HS** | **BV** | **UV** | **HS** | **BV** | **UV** | **HS** | **BV** | **UV** | **HS** |
| ***Stability*** | **ML CoM rom (mm)** | 2.16e+02 *(2.09e+02;3.79e+02)* | 2.55e+02 *(1.89e+02;3.29e+02)* | 1.45e+02 *(1.17e+02;2.15e+02)* |  | | |  | | |  | | |
|  |  | **η² = 0.234, p = 0.034* -** | | |  |  |  |  |  |  |  |  |  |
|  | **ML MoS (mm)** | 7.99e+01 *(6.32e+01;9.30e+01)* | 4.56e+01 *(3.14e+01;5.01e+01)* | 4.48e+01 *(3.98e+01;4.96e+01)* |  | | |  | | |  | | |
|  |  | ♦ **η² = 0.352, p = 0.006** (BV/HS : p = 0.022*; BV/UV : p = 0.008**)** | | |  |  |  |  |  |  |  |  |  |
|  | **AP MoS (mm)** | -5.85e+01 *(-8.72e+01;*  *-4.62e+01)* | -6.44e+01 *(-9.12e+01;*  *-2.88e+01)* | -6.14e+01 *(-1.04e+02;*  *-3.92e+01)* |  | | |  | | |  | | |
|  |  | η² = 0.007, p = 0.897 | | |  |  |  |  |  |  |  |  |  |
|  | **sag WBAM (ND)** | 6.90e-03 *(6.28e-03;7.65e-03)* | 7.06e-03 *(6.69e-03;.8.41e-03)* | 8.26e-03 *(7.74e-03;9.37e-03)* |  | | |  | | |  | | |
|  |  | η² = 0.169, p = 0.086 | | |  |  |  |  |  |  |  |  |  |
|  | **fro WBAM (ND)** | 6.89e-03 *(5.88e-03;7.10e-03)* | 7.26e-03 *(6.31e-03;7.84e-03)* | 7.11e-03 *(6.42e-03;8.73e-03)* |  | | |  | | |  | | |
|  |  | η² = 0.039, p = 0.570 | | |  |  |  |  |  |  |  |  |  |
|  | **cor WBAM (ND)** | 4.01e-03 *(3.41e-03;4.35e-03)* | 4.03e-03 *(3.33e-03;4.47e-03)* | 4.36e-03 *(3.84e-03;4.89e-03)* |  | | |  | | |  | | |
|  |  | η² = 0.040, p = 0.561 | | |  |  |  |  |  |  |  |  |  |
| ***Spatial*** | **CoM score (ND)** | 5.28e+02 *(4.72e+02;1.34e+03)* | 3.73e+02 *(3.52e+02;7.70e+02)* | 2.16e+02 *(1.46e+02;3.82e+02)* |  | | |  | | |  | | |
|  |  | **η² = 0.251, p = 0.026* (BV/HS : p = 0.023*)** | | |  |  |  |  |  |  |  |  |  |
|  | **Foot score (ND)** | 2.10e+03 *(1.65e+03;2.76e+03)* | 1.51e+03 *(9.47e+02;1.86e+03)* | 8.68e+02 *(5.88e+02;1.17e+03)* |  | | |  | | |  | | |
|  |  | ♦ **η² = 0.348, p = 0.006** (BV/HS : p = 0.004**)** | | |  |  |  |  |  |  |  |  |  |
|  | **Step width (mm)** | 1.55e+02 *(1.51e+02;1.92e+02)* | 1.31e+02 *(8.40e+01;1.60e+02)* | 9.04e+01 *(6.03e+01;1.13e+02)* |  | | |  | | |  | | |
|  |  | **η² = 0.300, p = 0.013* (BV/HS : p = 0.009**)** | | |  |  |  |  |  |  |  |  |  |
|  | **ML step distance (mm)** | 1.04e+02 *(8.38e+01;1.83e+02)* | 1.23e+02 *(1.06e+02;1.63e+02)* | 8.81e+01 *(5.64e+01;1.25e+02)* |  | | |  | | |  | | |
|  |  | η² = 0.122, p = 0.171 | | |  |  |  |  |  |  |  |  |  |
|  | **Step number (ND)** |  | | | 5.00e-01 *(0.00e+00;1.75e+00)* | 1.00e+01 *(1.00e+01;*  *1.00e+01)* | 1.00e+01 *(9.25e+00;*  *1.00e+01)* |  | | |  | | |
|  |  |  |  |  | ♦ **η² = 0.760, p < 0.001*** (BV/HS : p < 0.001***; BV/UV : p < 0.001***)** | | |  |  |  |  |  |  |
| ***Temporal*** | **Walking speed (m/s)** | 5.60e-01 *(4.21e-01;6.21e-01)* | 7.24e-01 *(6.46e-01;7.65e-01)* | 9.12e-01 *(7.70e-01;1.00e+00)* |  | | | 5.15e-01 *(3.27e-01;6.55e-01)* | 6.53e-01 *(5.38e-01;7.11e-01)* | 5.98e-01 *(5.45e-01;6.64e-01)* |  | | |
|  |  | ♦ **η² = 0.421, p = 0.002** (BV/HS : p = 0.002**)** | | |  |  |  | η² = 0.080, p = 0.312 | | |  |  |  |
|  | **Double support time (s)** | 3.45e-01 *(3.33e-01;4.23e-01)* | 3.60e-01 *(3.25e-01;4.11e-01)* | 3.30e-01 *(2.86e-01;3.63e-01)* |  | | |  | | |  | | |
|  |  | η² = 0.049, p = 0.490 | | |  |  |  |  |  |  |  |  |  |
|  | **Task time (s)** |  | | |  | | |  | | | 8.20e+00 *(7.52e+00;1.03e+01)* | 8.55e+00 *(7.98e+00;8.97e+00)* | 7.83e+00  *(7.34e+00;8.45e+00)* |
|  |  |  |  |  |  |  |  |  |  |  | η² = 0.065, p = 0.389 | | |
| ***Kinematic*** | **Head AI – Roll (ND)** | -2.67e-01 *(-3.18e-01;-4.25e-02)* | -3.28e-01 *(-3.99e-01;-1.54e-01)* | 1.24e-01 *(-1.25e-01;4.40e-01)* |  | | |  | | |  | | |
|  |  | ♦ **η² = 0.328, p = 0.009** (BV/HS : p = 0.036*; UV/HS : p = 0.011*)** | | |  |  |  |  |  |  |  |  |  |
|  | **Head AI – Pitch (ND)** | -2.76e-01 *(-6.53e-01;1.67e-01)* | -1.07e-01 *(-2.55e-01;4.53e-01)* | 4.37e-01 *(2.78e-01;6.96e-01)* |  | | |  | | |  | | |
|  |  | ♦ **η² = 0.323, p = 0.009** (BV/HS : p = 0.008**)** | | |  |  |  |  |  |  |  |  |  |
|  | **Head AI – Yaw (ND)** | -7.28e-01 *(-7.97e-01;-6.63e-01)* | -4.91e-01 *(-5.65e-01;-1.87e-01)* | -5.22e-02 *(-4.51e-01;4.67e-01)* |  | | |  | | |  | | |
|  |  | ♦ **η² = 0.349, p = 0.007** (BV/HS : p = 0.005**)** | | |  |  |  |  |  |  |  |  |  |
|  | **Head AV (rad/s)** | 2.37e-01 *(2.13e-01;2.83e-01)* | 2.20e-01 *(2.12e-01;2.50e-01)* | 2.43e-01 *(2.07e-01;2.69e-01)* |  | | |  | | |  | | |
|  |  | η² = 0.006, p = 0.910 | | |  |  |  |  |  |  |  |  |  |
|  | **Trunk AV (rad/s)** | 6.17e-01 *(5.22e-01;7.93e-01)* | 8.61e-01 *(6.83e-01;1.00e+00)* | 1.23e+00 *(7.87e-01;1.32e+00)* |  | | |  | | |  | | |
|  |  | **η² = 0.314, p = 0.011* (BV/HS : p = 0.010*)** | | |  |  |  |  |  |  |  |  |  |
|  | **GaitSD (°)** | 3.17e+00 *(2.89e+00;3.49e+00)* | 3.05e+00 *(2.45e+00;3.44e+00)* | 2.48e+00 *(2.08e+00;3.09e+00)* |  | | |  | | |  | | |
|  |  | η² = 0.082, p = 0.307 | | |  |  |  |  |  |  |  |  |  |

Supplementary Table 1- C. Median results (Q1;Q3) for all parameters calculated for each task, and results of Kruskal-Wallis test (alpha = 0.05), and in case of significant result, result of Dunn's post hoc test with Holm correction. *p ≤ 0.05; **p ≤ 0.01; ***p ≤ 0.001. Data for which the Kruskal-Wallis test, with alpha set at 0.01, is significant, are indicated by a ♦.
