# Supplementary figures and images for: Assessment of dynamic stability and identification of key tasks and parameters in patients with unilateral and bilateral vestibulopathy: a laboratory-based study
Source: Front Neurosci. 2025 Sep 8;19:1624948. doi: 10.3389/fnins.2025.1624948 (PMC12450868; doi:10.3389/fnins.2025.1624948)

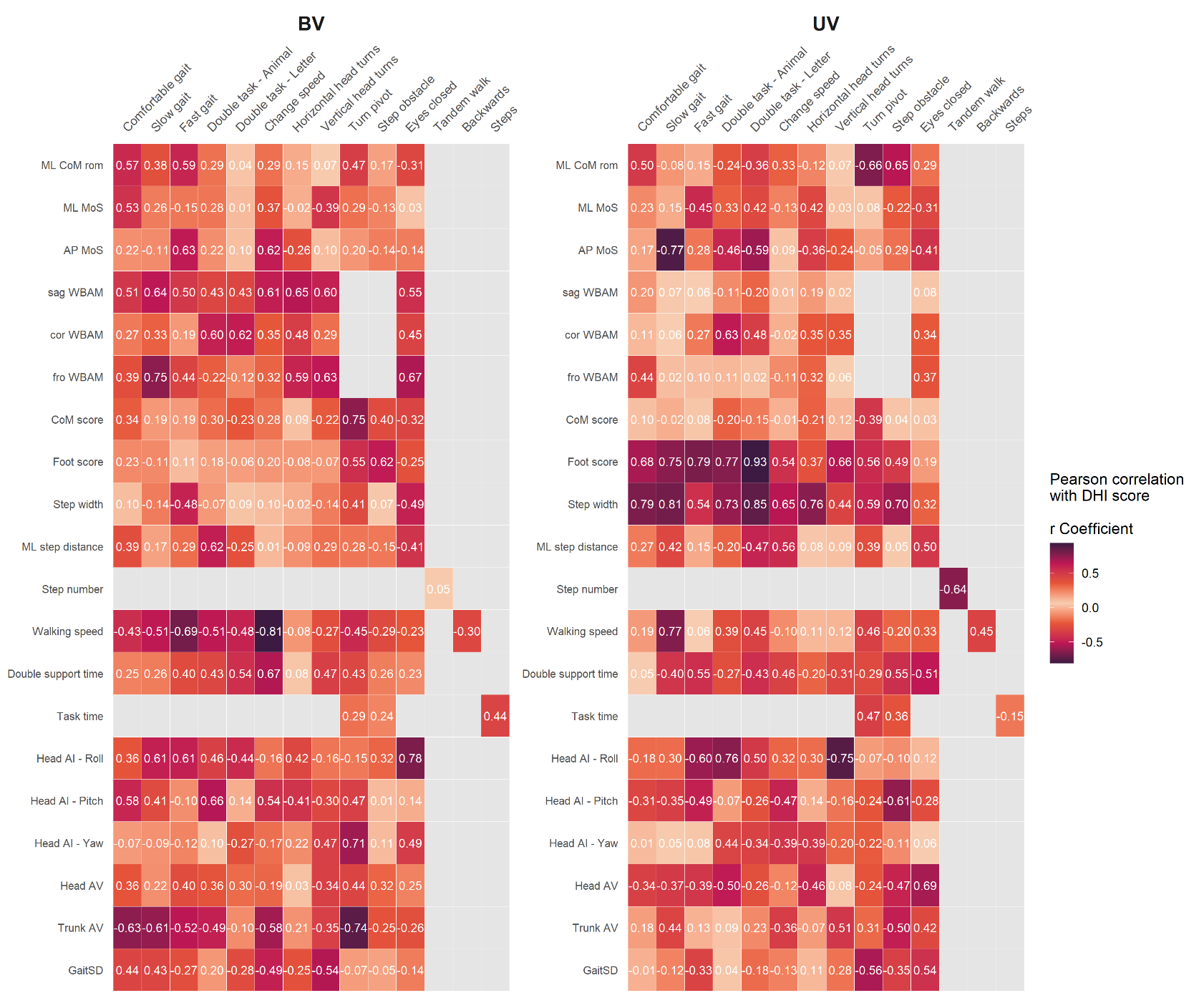


Supplementary Data 1. Correlation table (Pearson correlation) with DHI score.

Supplement: Supplementary file 2 [file Table_2.docx]
